# New Multiple Sclerosis Disease Severity Scale Predicts Future Accumulation of Disability

**DOI:** 10.3389/fneur.2017.00598

**Published:** 2017-11-10

**Authors:** Ann Marie Weideman, Christopher Barbour, Marco Aurelio Tapia-Maltos, Tan Tran, Kayla Jackson, Peter Kosa, Mika Komori, Alison Wichman, Kory Johnson, Mark Greenwood, Bibiana Bielekova

**Affiliations:** ^1^Neuroimmunological Diseases Unit, National Institute of Neurological Disorders and Stroke, National Institutes of Health, Bethesda, MD, United States; ^2^Department of Mathematical Sciences, Montana State University, Bozeman, MT, United States; ^3^PECEM, Facultad de Medicina, Universidad Nacional Autónoma de México, Mexico City, Mexico; ^4^Bioinformatics Section, National Institute of Neurological Disorders and Stroke, National Institutes of Health, Bethesda, MD, United States

**Keywords:** multiple sclerosis, clinical trials, neuroimmunology, neuroinflammation, clinical practice, education

## Abstract

The search for the genetic foundation of multiple sclerosis (MS) severity remains elusive. It is, in fact, controversial whether MS severity is a stable feature that predicts future disability progression. If MS severity is not stable, it is unlikely that genotype decisively determines disability progression. An alternative explanation tested here is that the apparent instability of MS severity is caused by inaccuracies of its current measurement. We applied statistical learning techniques to a 902 patient-years longitudinal cohort of MS patients, divided into training (*n* = 133) and validation (*n* = 68) sub-cohorts, to test four hypotheses: (1) there is intra-individual stability in the rate of accumulation of MS-related disability, which is also influenced by extrinsic factors. (2) Previous results from observational studies are negatively affected by the insensitive nature of the Expanded Disability Status Scale (EDSS). The EDSS-based MS Severity Score (MSSS) is further disadvantaged by the inability to reliably measure MS onset and, consequently, disease duration (DD). (3) Replacing EDSS with a sensitive scale, i.e., Combinatorial Weight-Adjusted Disability Score (CombiWISE), and substituting age for DD will significantly improve predictions of future accumulation of disability. (4) Adjusting measured disability for the efficacy of administered therapies and other relevant external features will further strengthen predictions of future MS course. The result is a MS disease severity scale (MS-DSS) derived by conceptual advancements of MSSS and a statistical learning method called gradient boosting machines (GBM). MS-DSS greatly outperforms MSSS and the recently developed Age Related MS Severity Score in predicting future disability progression. In an independent validation cohort, MS-DSS measured at the first clinic visit correlated significantly with subsequent therapy-adjusted progression slopes (*r* = 0.5448, *p* = 1.56e−06) measured by CombiWISE. To facilitate widespread use of MS-DSS, we developed a free, interactive web application that calculates all aspects of MS-DSS and its contributing scales from user-provided raw data. MS-DSS represents a much-needed tool for genotype-phenotype correlations, for identifying biological processes that underlie MS progression, and for aiding therapeutic decisions.

## Introduction

Multi-centric genetic consortia identified more than 200 multiple sclerosis (MS) susceptibility genes, but validated no gene variants associated with MS severity (1, 2). The aggressiveness of a patient’s MS course can be measured by the MS Severity Score (MSSS) (3), which relates Expanded Disability Status Scale (EDSS) (4) to MS disease duration (DD). A newer version of the EDSS-based MSSS, called the Age Related MS Severity Score (ARMSS), substitutes age for DD (5). However, it is unclear if MS severity represents a stable disease phenotype (6). Consequently, our ability to provide reliable prognostic information to patients is limited.

Large natural history studies have shown that past MS course does not predict future rate of disability accumulation once a patient reaches an EDSS of 6 (6, 7). This suggests that MS severity is either not a stable feature or represents a stable phenotype only for low-to-moderate disability levels. This understanding is essential because only stable features can be successfully used as outcomes for statistical learning techniques to identify genes, biomarkers, and/or biological processes that hasten MS progression.

Consequently, the purpose of this longitudinal study was to determine if MSSS (or ARMSS) can reliably predict future rates of disability accumulation measured by EDSS, and, if they cannot, to develop and validate a model that more reliably predicts MS severity for both clinical and research applications.

## Materials and Methods

### Patients

The study was approved by the Institutional Review Board and all patients signed an informed consent. Participants were selected from the prospectively acquired natural history protocol 09-N-0032 (Clinicaltrials.gov Identifier NCT00794352): “Comprehensive multimodal analysis of patients with neuroimmunological diseases of the CNS” based on the following criteria: (1) confirmed diagnosis of MS and (2) longitudinal follow-up consisting of three or more clinic visits with recorded CombiWISE values spanning ≥1 year or two or more clinic visits with recorded CombiWISE values spanning ≥3 years. We excluded clinical visits associated with MS exacerbation because these would overestimate the amount of sustained disability progression, as a large proportion of exacerbation-related disability is reversible. The mean length of follow-up was 4.49 ± 2.90 years. All MS subjects fulfilling these criteria as of 5/26/2017 were included in this analysis. Pertinent inclusion criteria for 09-N-0032 protocol are (1) presentation with a clinical syndrome consistent with an immune-mediated central nervous system (CNS) disorder, (2) neuroimaging evidence of inflammatory and/or demyelinating CNS disease, and (3) 12–75 years of age at the time of enrollment. The final diagnosis was assigned after thorough diagnostic work-up as described (8). Patient demographics and other clinical characteristics are provided in Table 1.

**Table 1:** Patient demographics and other clinical characteristics.

	Training (*N* = 133)	Validation (*N* = 68)	Total (*N* = 201)
**Demographics**			

**Age (years)**			
Mean ± SD	48.5 ± 12.0	49.0 ± 10.9	48.6 ± 11.6
Median	49.2	49.9	49.5
Range	24.0–67.8	23.2–70.3	23.2–70.3
**Gender, no. of patients (%)**			
Female	66 (49.6)	34 (50.0)	100 (49.8)
Male	67 (50.4)	34 (50.0)	101 (50.2)
**Race, no. of patients (%)**			
White	116 (87.2)	60 (88.2)	176 (87.6)
Other	17 (12.8)	8 (11.8)	25 (12.4)
**Smoking history, no. of patients (%)**			
Yes	80 (60.2)	41 (60.3)	121 (60.2)
No/unknown	53 (39.8)	27 (39.7)	80 (39.8)
**Family history of multiple sclerosis (MS), no. of patients (%)**			
No	87 (65.4)	48 (70.6)	135 (67.2)
Mild (1 distant relative with MS)	22 (16.5)	6 (8.8)	28 (13.9)
Moderate (>1 distant relative with MS)	7 (5.3)	3 (4.4)	10 (5.0)
Strong (first degree relative with definite MS)	10 (7.5)	6 (8.8)	16 (8.0)
Unknown	7 (5.3)	5 (7.4)	12 (6.0)

**Other clinical characteristics**			

Age at disease onset (years)			
Mean ± SD	36.5 ± 11.6	38.5 ± 11.1	37.1 ± 11.4
Median	35.9	39.4	37.2
Range	7.9–63.7	16.5–60.1	7.9–63.7
**Disease duration (years)**			
Mean ± SD	12.0 ± 10.2	10.5 ± 9.2	11.5 ± 9.9
Median	9.4	7.7	8.7
Range	0.1–39.5	0.1–42.3	0.1–42.3
**EDSS**			
Mean ± SD	4.0 ± 2.3	3.9 ± 2.2	3.9 ± 2.2
Median	4	3	4
Range	0.0–7.5	0–6.5	0.0–7.5
**MSSS**			
Mean ± SD	5.1 ± 2.6	5.2 ± 2.4	5.1 ± 2.5
Median	5.6	5.0	5.4
Range	0.1–9.8	0.8–9.7	0.1–9.8
**CombiWISE**			
Mean ± SD	31.0 ± 18.3	29.2 ± 16.0	30.4 ± 17.5
Median	32.7	25.9	30.2
Range	1.6–79.5	3.9–53.1	1.6–79.5
COMRIS-CTD*			
Mean ± SD	12.9 ± 6.5	12.7 ± 6.1	12.8 ± 6.3
Median	12.8	12.9	12.9
Range	0.0–29.9	2.1–28.5	0.0–29.9

Data are mean ± SD, median, and range or *n* (%). Characteristics labeled with an (*) were calculated by excluding missing data. Percentages may not sum to 100 due to rounding.

*EDSS, Expanded Disability Status Scale; MSSS, MS Severity Score; CombiWISE, Combinatorial Weight-Adjusted Disability Score; COMRIS-CTD, Combinatorial MRI Score of CNS tissue destruction*.

### Data Collection

All data were prospectively acquired and entered into the research database. Clinicians acquired the EDSS (4) and Scripps Neurological Rating Scale (SNRS) (9), while a different set of investigators acquired the Timed 25-Foot Walk (T25FW) and 9-Hole Peg Test (9HPT) scores. From these data, the research database automatically computed the CombiWISE score based on the published formula (10):

(1) CombiWISE=33.166+3.803(EDSS)−0.407(SNRS)+2.409(log2(T25FW))+18.056(T25FWFAIL)+1.305(log2(NDH-9HPT))+10.751(NDHFAIL),

where log_2_(T25FW) is the logarithm (base 2) of the T25FW, T25FW_FAIL_ is binary (0 for completed test, 1 for failed test), log_2_(NDH-9HPT) is the logarithm (base 2) of the non-dominant hand 9HPT, and NDH_FAIL_ is binary (0 for completed test, 1 for failed test). Prospectively acquired MRI scans were semi-quantitatively rated according to the published protocol (11) and grades were entered into the research database, which automatically calculates Combinatorial MRI Scale of CNS Tissue Destruction (COMRIS-CTD) as described (11).

While current disease modifying therapies (DMTs) were entered into the database prospectively at the time of each clinical visit, detailed history of *past* DMTs, smoking, and family history were gathered from prospectively acquired patient questionnaires and clinic notes. When such detailed history data were missing, an investigator obtained them retrospectively over the phone or during a follow-up clinic visit. All data underwent weekly quality control (QC), after which the data were locked in the database.

For analysis, QC’d data were exported into Excel and then imported into the statistical software R v3.3.1 (RStudio v1.0) (12, 13). To ensure that both training and validation cohorts were representative of the underlying population, we used the following methods to construct training and validation datasets: patients were first separated based on their race (Caucasian and non-Caucasian) and gender (male and female). Within each of these four groups, patients were binned according to whether they were above or below the median measurements for therapy-adjusted CombiWISE/age (derived from data collected at the first clinic visit; see definition later in Section “Materials and Methods”) and age at first clinic visit. Within each of these 12 groupings, approximately two-thirds of patients were randomly selected as a training dataset, leaving the remaining one-third of patients as a validation dataset. This procedure balanced the distribution of the main demographic features and disability between the training and validation cohorts.

### Adjusting for Therapeutic Efficacy

In a previous study (14), we performed a meta-analysis of 38 randomized, blinded clinical trials of immunomodulatory therapies in MS to test the hypothesis that efficacy of current immunomodulatory DMTs on disability progression decreases with age. After fitting a weighted regression with an interaction term, treatment efficacy was found to be highly associated with age, with differences in outcome (inhibition of disability progression) dependent on drug classification into high- or low-efficacy categories [*R*^2^ (coefficient of determination) = 0.6757, *p*-value for interaction <0.0001].

Therefore, to adjust measured disability values (i.e., CombiWISE) and their longitudinal change for the *average* age-adjusted therapeutic efficacy of administered DMTs, we used the weighted linear regression from the previously described meta-analysis. We used the following strategies for adjustment: 1. cross-sectional disability data collected at first clinic visit and utilized for predicting future disability course were adjusted for all past treatments received before the first National Institutes of Health (NIH) clinic visit. We applied an identical strategy for adjusting cross-sectional disability data measured at the last clinic visit to determine whether MS disease severity scale (MS-DSS) at the last clinic visit also correlated with measured longitudinal data. 2. We then measured progression slopes derived from disability measurements obtained at all visits—this represented the “future” disability progression we wanted to predict from single-visit data. Because most patients were treated during this longitudinal follow-up, we also had to adjust these “future” progression slopes for *de facto* “future” treatments. Mathematically, the process of adjustment for therapeutic efficacy can be described as follows.

First, we calculated the cumulative efficacy across the entire treatment period of duration *t_n_* − *t*_0_, using the following formula:

(2) $$\begin{array}{l} \text{Eff}(t_{0},t_{n})=\frac{\left(t_{1}-t_{0}){\text{Eff}}_{\text{1}}(t_{1},t_{0})+(t_{2}-t_{1}){\text{Eff}}_{2}(t_{2},t_{1})+\ldots \;+(t_{n}-t_{n-1}){\text{Eff}}_{n}(t_{n},t_{n-1}\right)}{t_{n}-t_{0}}\\ =\sum_{i=1}^{n}\frac{\left(t_{i}-t_{i-1}){\text{Eff}}_{i}(t_{i},t_{i-1}\right)}{t_{n}-t_{0}}, \end{array}$$

where *t*_0_ is the age at the first visit, *t_n_* is the age at the last visit, and each term Eff*_i_* represents therapeutic efficacy generated from the following linear models:

(3) $$\text{Untreated/Unknown Efficacy}:\;{\text{Eff}}_{\text{i}}(t_{i},t_{i}{\;}_{-1})=0,$$

(4) $$\text{Low-Efficacy}:\;{\text{Eff}}_{i}(t_{i},t_{i-1})=-1.50\left(\frac{t_{i}+t_{i-1}}{2}\right)+83.71,$$

(5) $$\text{High-Efficacy}:\;{\text{Eff}}_{i}(t_{i},t_{i-1})=-4.34\left(\frac{t_{i}+t_{i-1}}{2}\right)+206.39,$$

where the term (*t_i_* + *t_i_*_-1_)/2 is an average of two time points. The dichotomization of drugs into low- versus high-efficacy was described in the meta-analysis (14) and will be also mentioned in the results. All negative values for efficacy were assumed to be 0.

Measured progression slopes may be positive (i.e., patient accumulates disability) or negative (i.e., patient is repairing previously accumulated disability). If a patient remained untreated, then the measured progression slope quantifies the true progression slope. However, if a treated patient continued to accumulate clinical disability, then the true progression without therapy would be equivalent to shifting the measured slope upward by a magnitude that corresponds to the efficacy of the administered DMT [i.e., Eff(*t*_0_, *t_n_*)]. This, in effect, increases the rate of progression to provide an estimate of expected disease accumulation without therapy. Therefore, adjusted CombiWISE slopes were calculated by *adding* the measured CombiWISE slopes (fit by simple linear regression) to the percent adjustment due to efficacy, as follows:

(6) $$\text{Adjusted CW Slope }=\text{ Measured CW Slope }+\;(\text{Adjusted CW Slope})\cdot \text{Eff}(t_{0},t_{n}).$$

Solving for the adjusted CombiWISE slope gives

(7) $$\text{Adjusted CWSlope}=\frac{\text{Measured CW Slope}}{\left(1-\text{Eff}(t_{0},t_{n})\right)}.$$

For patients who improved while taking DMT, the true progression without treatment would be equivalent to shifting the negative measured slope upward by a magnitude, Eff(*t*_0_, *t_n_*), of the absolute value of the measured slope. Since the slope is negative, this is equivalent to subtracting the product of the measured slope and efficacy and, in effect, decreases the expected rate of improvement to model disease accumulation without therapy. Therefore, the adjusted CombiWISE slopes were similarly calculated by *subtracting* the percent adjustment due to efficacy from the measured CombiWISE slopes, as follows:

(8) $$\text{Adjusted CW Slope }=\text{ Measured CW Slope }-\;(\text{Measured CW Slope})\cdot \text{Eff}(t_{0},t_{n}).$$

Factoring the right-hand side gives

(9) $$\text{Adjusted CW Slope }=\text{ Measured CW Slope}\cdot \left(1-\text{Eff}(t_{0},t_{n})\right).$$

We, then, adjusted the first measured CombiWISE, CW_measured_ (*t*_0_), and last measured CombiWISE, CW_measured_ (*t_n_*), upward, as follows:

(10) $${\text{CW}}_{\text{adjusted}}(t_{0})=\frac{{\text{CW}}_{\text{measured}}(t_{0})}{\left(1-\text{Eff}(0,t_{0})\right)},$$

and

(11) $${\text{CW}}_{\text{adjusted}}(t_{n})=\frac{{\text{CW}}_{\text{measured}}(t_{n})}{\left(1-\text{Eff}(0,t_{n})\right)},$$

where Eff(0, *t*_0_) represents the total cumulative efficacy from birth to the first clinic visit, and Eff(0, *t_n_*) represents the total cumulative efficacy from birth to the last clinic visit. Functions within the R script were used to generate all therapy adjustments. The Supplementary Material contains the utilized R code in its entirety.

### Optimization of MS-DSS by Statistical Learning

A Gradient Boosting Machine [GBM (15, 16)] was used to develop a model predicting therapy-adjusted CombiWISE Slopes from baseline covariates collected during a patient’s initial visit. GBM’s are sequentially built using regression trees. The current tree is built using residuals from the previous tree’s predictions, and the predictions are iteratively updated by adding the current tree’s prediction (times a shrinkage parameter between 0 and 1) to the previous tree’s prediction. For each tree constructed, a random sample containing half of the observations is withheld from the training cohort to introduce randomness into the modeling process (called the out-of-bag or OOB sample). The main tuning parameters for a GBM are the depth of the individual trees (sometimes called the interaction depth, typically between 1 and 4), the shrinkage parameter (typically small, less than 0.1), and the number of trees. Using the gbm R package (17), we selected an interaction depth of 2, a conservative shrinkage parameter of 0.001, and used a five-fold cross validation to select the number of trees. The relative influence of each of the variables was computed by examining the improvement in squared-error from splits within each individual tree and averaging these improvements across all trees in the ensemble.

## Results

### MSSS and ARMSS Do Not Predict Development of Future Disability Measured with EDSS

We calculated patient-specific linear regression slopes (“progression slopes”) from clinical scales (EDSS and CombiWISE) collected at each clinic visit. EDSS measured during the first clinic visit was then used to calculate MSSS (3) and ARMSS (5) as described.

**Figure 1:**
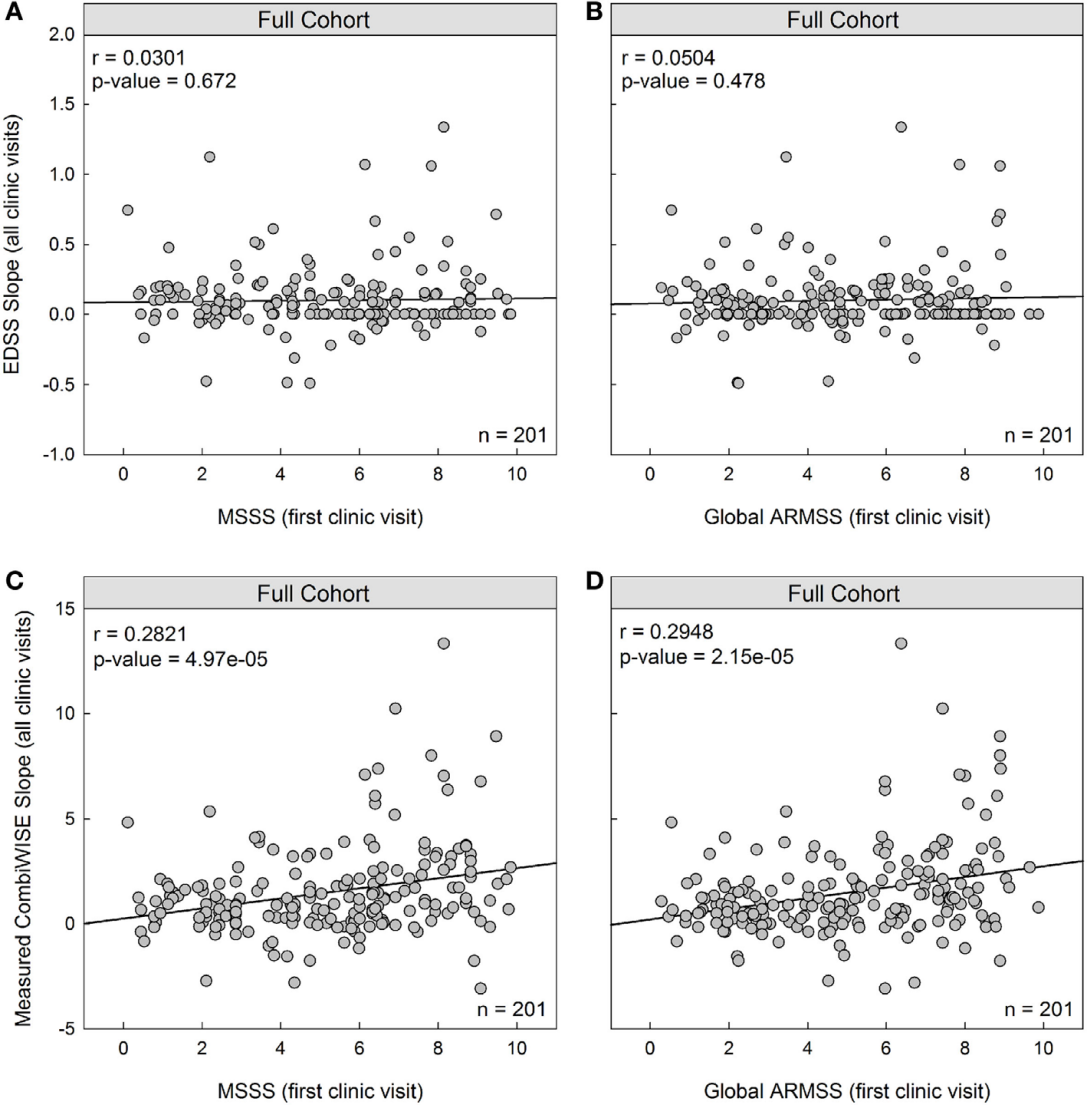
MS Severity Score (MSSS) and Age Related MS Severity Score (ARMSS) predict development of future disability measured with CombiWISE slope, but not Expanded Disability Status Scale (EDSS) slope. **(A,B)** Cross-sectional MSSS and ARMSS measured at the first clinic visit do not predict future accumulation of disability measured by EDSS regression slopes. **(C,D)** Cross-sectional MSSS and ARMSS measured at the first clinic visit correlate with longitudinal CombiWISE regression slopes derived from subsequent longitudinal follow-up. MSSS, ARMSS, and CombiWISE values were calculated according to published formulas (3, 5, 10), and subsequent EDSS and CombiWISE regression slopes were derived using least squares fitting (see Materials and Methods). Patients were prospectively followed for an average of 4.49 years. Correlations are shown for the entire cohort (*n* = 201).

To determine whether MSSS predicts future disease severity measured by EDSS progression slopes, we assessed the correlation between patient-specific MSSS derived from first visit data and EDSS progression slopes derived from subsequent longitudinal follow-up. The resulting correlation in the full cohort (*n* = 201) was poor and not statistically significant (*r* = 0.0301, *p* = 0.672; Figure 1A). A poor predictive result was also observed for ARMSS, which ranks EDSS based on age (*r* = 0.0504, *p* = 0.478; Figure 1B).

We hypothesized that these null results were due to the insensitivity of EDSS to correctly measure MS progression in intervals shorter than 10 years. To test this hypothesis, we investigated whether MSSS and ARMSS predict future progression slopes using a more sensitive and continuous scale, CombiWISE (10). We found a statistically significant, albeit weak, correlation between both MSSS and measured CombiWISE progression slopes (*r* = 0.2821, *p* = 4.97e−05; Figure 1C) and ARMSS and measured CombiWISE progression slopes (*r* = 0.2948, *p* = 2.15e−05; Figure 1D).

### CombiWISE Progression Slopes Explain a Larger Proportion of Variance than EDSS Progression Slopes

**Figure 2:**
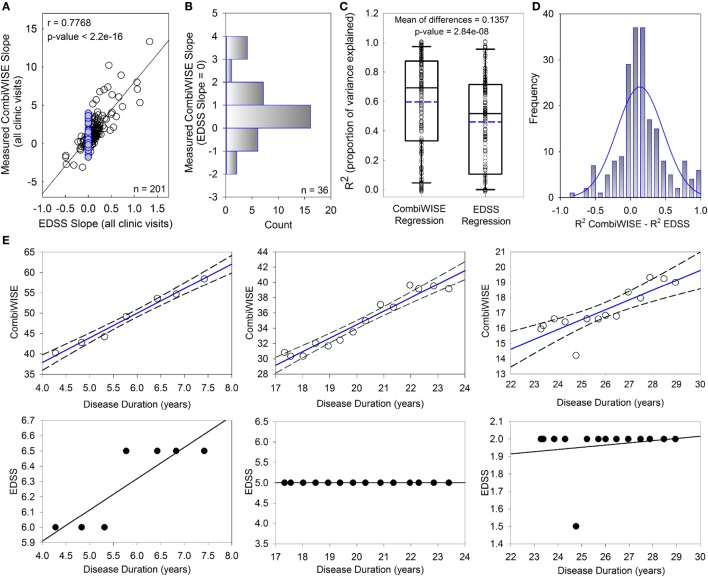
CombiWISE measures multiple sclerosis (MS) progression more accurately than Expanded Disability Status Scale (EDSS). **(A)** Patient-specific progression rates measured by EDSS and CombiWISE regression slopes strongly correlate. **(B)** Frequency distribution of measured CombiWISE slopes for patients with no reported change in EDSS [highlighted blue points in **(A)**]. The histogram demonstrates the insensitivity of EDSS; patients can progress as much as 4 CombiWISE units/year (or improve by up to 2 CombiWISE units/year) with no change in EDSS. **(C)** Boxplots depict the proportion of variance explained for CombiWISE versus EDSS regressions. The median is indicated with a solid black line, and the mean is indicated with a dashed blue line. The bottom and top of the box denotes the first and third quartiles, respectively. The whiskers indicate the 10th and 90th percentiles. A two-tailed paired *t*-test indicated strong evidence of a mean difference of 0.1357 (95% CI, 0.089–0.182) in *R*^2^ values between the CombiWISE and EDSS regressions. **(D)** Histogram of difference in *R*^2^ values between CombiWISE and EDSS regressions suggests that the values are relatively normally distributed. **(E)** Representative patient examples illustrate higher accuracy of linear regressions (solid blue lines with dashed 95% CI; upper panels) derived from longitudinal CombiWISE measurements (no fill circles; upper panels) in comparison to linear regressions (solid black lines; lower panels) derived from longitudinal EDSS measurements (black filled circles; lower panels) from the same three MS patients.

While EDSS and CombiWISE progression slopes are strongly correlated (*r* = 0.7768, *p* < 2.2e−16; Figure 2A), most patients with EDSS progression slopes of 0 (i.e., EDSS “stable” patients highlighted in blue) had non-zero CombiWISE progression slopes (Figure 2B). Of these patients, two-thirds progressed in CombiWISE, as demonstrated by the positively skewed distribution of CombiWISE progression slopes for patients with unchanged EDSS (Figure 2B).

The proportion of variance explained (*R*^2^) reflects how well the regression lines, as a function of time, explain the measured responses for each patient. The full cohort data (Figure 2C) demonstrated unequivocal superiority of CombiWISE over EDSS in its ability to reliably measure MS disability progression slopes. A higher proportion of variance was explained on average for CombiWISE than for EDSS (Figure 2C; mean of differences = 0.1357, 95% CI = [0.089, 0.182], *p* = 2.84e−08). Figure 2D shows the distribution of the pairwise differences, reinforcing the improved representation of the changes over time possible when using CombiWISE as a response over EDSS. Representative examples of individual patients (Figure 2E) provide visual clues as to why CombiWISE is a better scale. In the second example, a representative patient with stable disease based on EDSS (i.e., EDSS slope of 0) had a clear linear progression of disability measured by CombiWISE (Figure 2E, middle panels).

### Substituting Measured CombiWISE/Age for MSSS Improves Predictive Power

It is impossible to measure MS onset and, therefore, disease duration (DD), with high precision. Since, in natural history cohorts, MS patients reached major disability milestones around the same age (and not around the same DD) (6), we hypothesized that replacing DD with age and replacing EDSS with CombiWISE would further strengthen predictions of future progression rates. The same hypothesis led previously to ARMSS, which demonstrated comparable, but not superior, power to detect disability differences when compared to MSSS (5).

**Figure 3:**
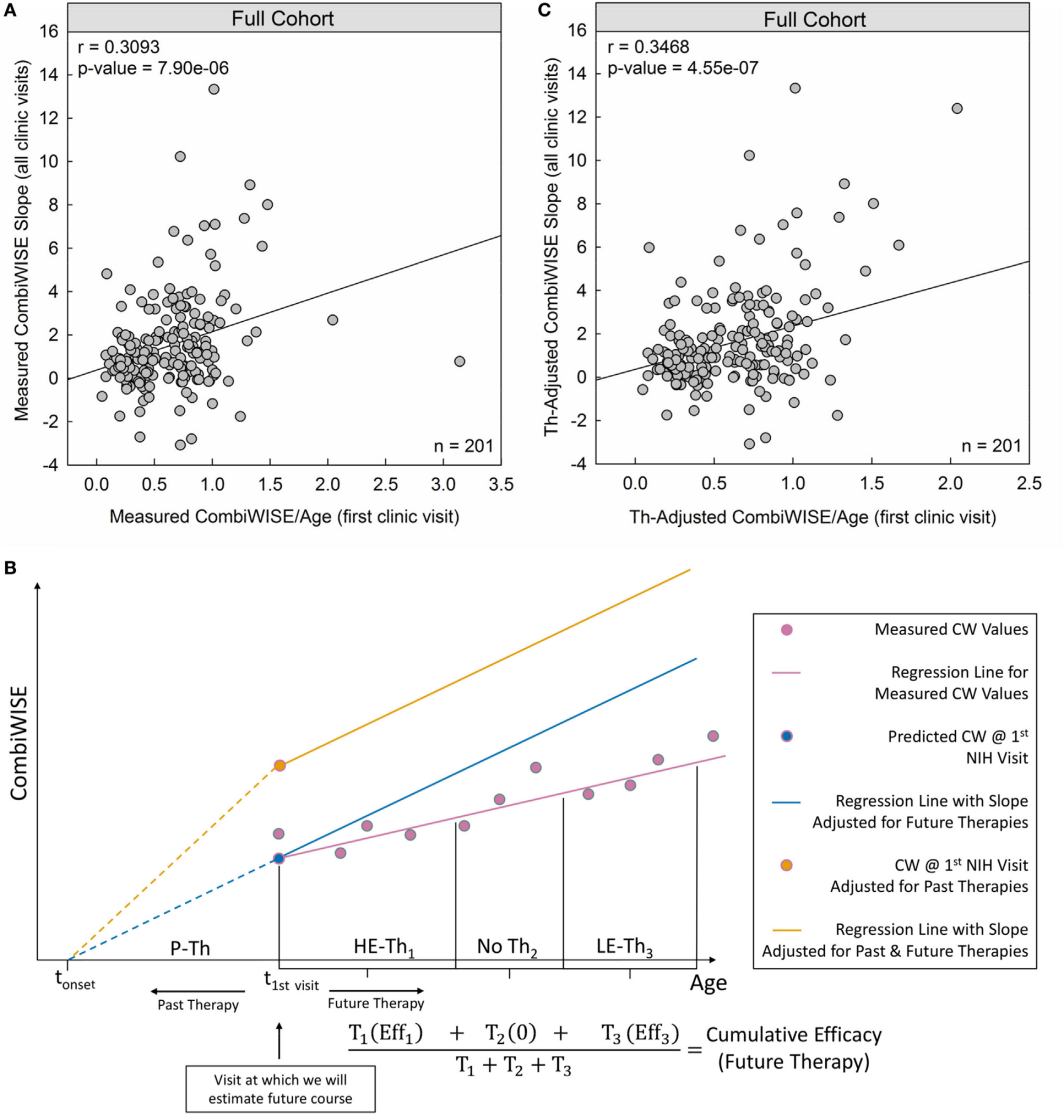
Therapy-adjusted CombiWISE/age is a better predictor of future disability than measured CombiWISE/age. **(A)** Patient-specific CombiWISE progression slopes moderately correlate with cross-sectional CombiWISE/age measured at the first clinic visit. CombiWISE values were calculated based on a published formula (10), and subsequent regression slopes were derived using least squares fitting from an average follow-up of 4.49 years. **(B)** In this hypothetical example, the patient began the multiple sclerosis disease process at time *t*_onset_ and was treated with past therapy (P-Th) prior to his/her first clinic visit at *t*_1st visit_. During this visit, we collected data used for estimating future disease course (i.e., MS disease severity scale). During subsequent longitudinal follow-up, this patient received high-efficacy therapy for duration T_1_. S/he then remained untreated for duration T_2_ before beginning low-efficacy therapy (LE-Th_3_) for duration T_3_. The mean-therapeutic efficacy (see Materials and Methods) for each treatment period was calculated as a function of the average age within that period and then weighted by the treatment duration. Any untreated periods were assumed to have zero efficacy so that the sum of all weighted products was unchanged by periods of no treatment. The final cumulative efficacy during longitudinal follow-up was then calculated as the ratio of this sum to the follow-up duration (T_1_ + T_2_ + T_3_). The slope of the measured CW regression (pink line) was adjusted by this patient-specific cumulative efficacy to derive an adjusted slope for future therapies (slope of blue line). For example, if the cumulative efficacy during longitudinal follow-up was 30%, the slope of the regression line of measured CW values would represent 70% of the new therapy-adjusted slope. An analogous adjustment was made for the sum of past treatments to derive the yellow point (CombiWISE at first clinic visit adjusted for all past therapies). The yellow line depicts the patient’s disease progression had s/he remained untreated (i.e., no past or future therapies). This line has the same slope as the blue line, but a higher intercept at *t*_1st visit_ due to adjustments for the cumulative efficacy of all past therapies. **(C)** After adjusting both variables for the effects of administered therapy (see Materials and Methods), the correlation between cross-sectional CombiWISE/age and longitudinal progression slopes improved.

Indeed, measured CombiWISE/age outperformed both MSSS and ARMSS in predicting future CombiWISE progression [MSSS: *r* = 0.2821, *p* = 4.97e−05 (Figure 1C); ARMSS: *r* = 0.2948, *p* = 2.15e−05 (Figure 1D); measured CombiWISE/age: *r* = 0.3093, *p* = 7.90e−06 (Figure 3A)].

### Adjusting Disability Measures for the Effects of Administered Treatments

Although using cross-sectional CombiWISE/age values strengthened correlations with subsequent CombiWISE slopes, this model could still explain only a small proportion of the variance in these slopes. Therefore, we sought other sources of error that could potentially be eliminated. One apparent source of error involves disregarding the effects of DMTs, as clinical trials convincingly demonstrated that (at least some) DMTs inhibit rates of disability progression.

We recently performed a meta-analysis of all randomized, blinded clinical trials (38 trials, >28,000 MS patients) of immunomodulatory MS drugs that reported efficacy on disability progression (14) to test the hypothesis that age has a major effect on the efficacy of administered DMTs. This meta-analysis confirmed that the efficacy of these drugs decreases as a patient ages, with age alone explaining approximately 42% of the variance seen in drug efficacy. We fit a regression, weighted for sample size and trial duration, to clinical trial data in FDA-approved drugs (for approved indications) and treated this regression as the average patient on an average DMT. Within each drug type, we also averaged the weighted residuals (difference between the observed and predicted efficacies) from all clinical trials of that particular drug and used the sign of this average to dichotomize the groups. Drugs for which the average weighted residuals were below the regression line (i.e., negative averages of weighted residuals from all clinical trials of the particular drug, implying that this drug has lower than average efficacy of all drugs combined) were classified as low-efficacy (glatiramer acetate, teriflunomide, all interferon beta preparations, dimethyl fumarate, and fingolimod). Analogously, drugs with average weighted residuals above the regression line (i.e., positive averages of weighted residuals from all clinical trials of the particular drug, implying that this drug has higher than average efficacy of all drugs combined) were classified as high-efficacy (natalizumab, daclizumab, alemtuzumab, mitoxantrone, and ocrelizumab). We used this dichotomization and a step-down testing procedure to arrive at a model that included the interaction between age and efficacy categorization (0 for low and 1 for high). This model was an improvement over the previous regression only on age (*R*^2^ = 0.6757 versus *R*^2^ = 0.4163, *t*-test = −3.464 for interaction on 36 DF, *p* = 0.002) and justified exploring the two efficacy categorizations separately, to produce the models summarized in Section “Materials and Methods” (Eqs 3–5). We used these regressions to adjust patient-specific cross-sectional CombiWISE values measured at the first and last clinic visit for all prior treatments. Analogously, we adjusted raw CombiWISE progression slopes for all treatments received during longitudinal follow-up. These adjustments are described in the next section.

### Therapy-Adjusted CombiWISE/Age Better Predicts Therapy-Adjusted CombiWISE Slopes

Conceptually, to adjust measured CombiWISE progression slopes, we divided each treatment period into therapeutically homogeneous segments. For example, if a patient received two treatments separated by a period of no therapy, the longitudinal follow-up would be divided into three segments (Figure 3B). We computed the patient’s mean age as an average of the initiation and termination ages within each treatment period and then used this age as input for Eqs 3–5. For untreated patients, the therapeutic efficacy was assumed to be 0 (Eq. 3). By averaging the therapeutic efficacies within each treatment period proportional to the duration of each treatment period (Figure 3B), we derived a cumulative efficacy for the entire longitudinal follow-up. The measured progression slope was then adjusted by this patient-specific cumulative efficacy to derive a “therapy-adjusted CombiWISE Slope,” which represents a predicted progression slope had the patient remained untreated.

Similarly, to adjust cross-sectional CombiWISE scores obtained at the first or last clinic visit for all preceding treatments, we calculated the cumulative efficacy of past treatments. Because exact MS onset cannot be determined for most patients, we used time from birth to the time of cross-sectional visit for these adjustments.

Using therapy-adjusted data improved the correlation between cross-sectional CombiWISE/age and subsequent longitudinal progression slopes from measured values (*r* = 0.3093, *p* = 7.90e−06; Figure 3A) to therapy-adjusted values (*r* = 0.3468, *p* = 4.55e−07; Figure 3C).

### Using Statistical Learning with Known and Newly Developed Variables to Derive Optimized MS-DSS Model

Finally, we asked whether we could further enhance the predictive power of future disability progression from a single cross-sectional measurement by using unbiased statistical learning. Because statistical learning uses measured data to obtain new knowledge, the validity of a model derived from such learning is best assessed on a new dataset not utilized for the modeling. This ensures that the model is generalizable to an entire population and not limited to the sample on which the model was trained. Thus, we split the cohort of 201 MS patients into training (*n* = 133) and independent validation (*n* = 68) cohorts using stratification based on therapy-adjusted CombiWISE/age (at first visit), age (at first visit), race, and gender to ensure that both training and validation cohorts were representative of the underlying population.

In addition to variables that were previously hypothesized or shown to affect disability progression such as gender, smoking, and race, we selected new features for statistical learning based on clinical knowledge. We hypothesized that the following features may negatively influence recovery from CNS lesions and, therefore, speed up accumulation of disability: age and the amount of CNS tissue destruction reflected by baseline disability (measured by CombiWISE) and/or quantified by MRI using published Combinatorial MRI Scale of CNS Tissue Destruction [COMRIS-CTD (11)]. Both factors diminish available reserves for restoring lost function by plasticity, while aging limits both plasticity and remyelination. Although MS susceptibility alleles do not seem to strongly affect MS severity (1, 2), we wanted to formally test the contribution of family history of MS in our model. Finally, we tested new features related to therapy: (1) number of DMTs taken for less than 6 months. We hypothesized that this feature reflects either side-effects or lack of therapeutic efficacy and, therefore, may positively correlate with MS severity. (2) Difference between therapy-adjusted and measured CombiWISE at the first clinic visit. We hypothesized that patients who benefited strongly from previous treatments may have better-than-average benefit from subsequent treatments. (3) Delay between onset of neurological symptoms attributable to MS and initiation of therapy. Since compartmentalization of MS inflammation is a continuous process that starts at MS initiation (18) and because compartmentalized inflammation is inadequately targeted by current DMTs (19), we hypothesized that delay in initiation of effective therapy will enhance the rate of disability accumulation, (4) the cumulative efficacy of therapy from first to last visit, and (5) the type of therapy received (i.e., none/unknown, low only, high only or both). While these final two features are, *de facto*, contained in our mathematical adjustment for the efficacy of administered therapies, we included them in the model to test whether these independent features provide any additional information not captured by linear regression(s).

We selected GBM for statistical learning, which can handle observations with missing predictors, and are known for their excellent predictive ability (15, 16). They can also effectively deal with interactions between selected features, which are likely to occur in the MS-DSS model.

**Figure 4:**
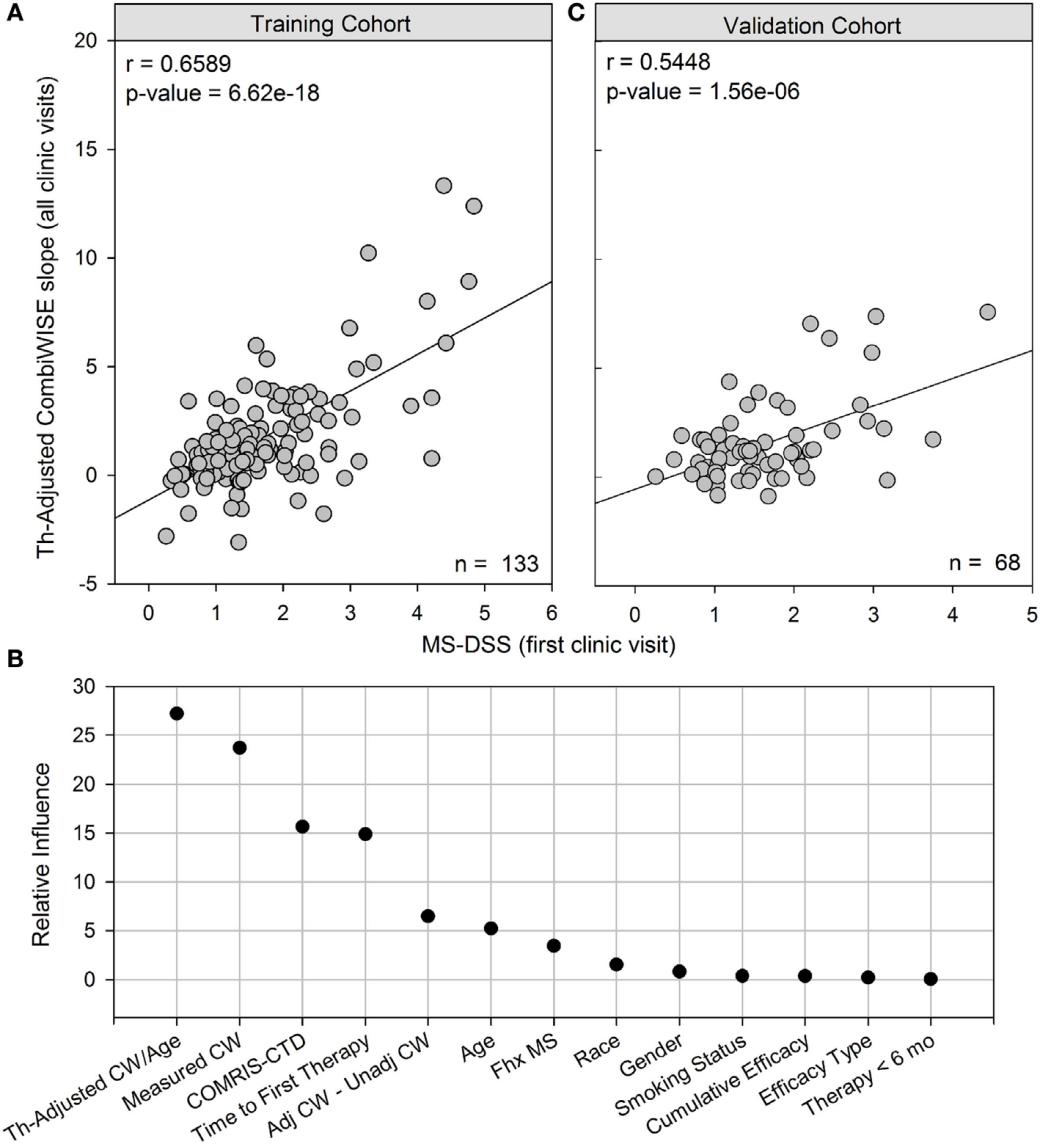
MS disease severity scale (MS-DSS) (at first clinic visit) is a strong predictor of future disability measured with therapy-adjusted CombiWISE slopes. **(A)** Cross-sectional MS-DSS measured at the first clinic visit strongly correlates with longitudinal therapy-adjusted CombiWISE regression slopes in the training cohort. **(B)** Relative influence of individual features in MS-DSS model. The Gradient Boosting Machine assigned importance to the following variables measured at the first clinic visit: therapy-adjusted CombiWISE/age, measured CombiWISE, COMRIS-CTD, time from disease onset to first therapy, difference between adjusted CombiWISE and unadjusted CombiWISE, age, and family history of MS. Known dichotomous classifiers of MS such as gender, race (Caucasian or non-Caucasian), and smoking history were determined to have little if any influence on model results. **(C)** Cross-sectional MS-DSS measured at the first clinic visit also strongly correlates with longitudinal therapy-adjusted CombiWISE regression slopes in the validation cohort. CombiWISE values were calculated based on a published formula (10), and subsequent therapy-adjusted CombiWISE slopes were derived using least squares fitting (see Materials and Methods) for patients with an average follow-up of 4.49 years.

The GBM model greatly outperformed simple linear regression based on therapy-adjusted CombiWISE/age, achieving a correlation of *r* = 0.6589, *p* = 6.62e−18 in the training cohort (Figure 4A).

As demonstrated in Figure 4B, the GBM trained on a training cohort (*n* = 133) assigned higher influence to new variables, such as measured CombiWISE, COMRIS-CTD, and difference between adjusted CombiWISE and unadjusted CombiWISE, than to known dichotomous modifiers of MS disease course, such as gender, smoking, or race. Family history of MS contributed only marginally to the MS-DSS model. Although age also had only marginal importance in the model, one should not forget that age is also present in the most influential variable in the model, therapy-adjusted CombiWISE/age, and in the mathematical adjustments of administered treatments. To simplify the MS-DSS model, we retrained the GBM model on a reduced set of covariates, including all variables determined to have relative influence above 4.0 and excluding all those with little to no relative influence. The final reduced model included the following list of variables, all measured at the first clinic visit: therapy-adjusted CombiWISE/age, measured CombiWISE, COMRIS-CTD, time from disease onset to first therapy, difference between adjusted CombiWISE and unadjusted CombiWISE, age, and family history of MS. This final model achieved a correlation of *r* = 0.5448, *p* = 1.56e−06 in an independent validation cohort (Figure 4C).

### Data Required to Compute MS-DSS Can Be Obtained *Post Hoc* and Used to Accurately Predict MS Severity

**Figure 5:**
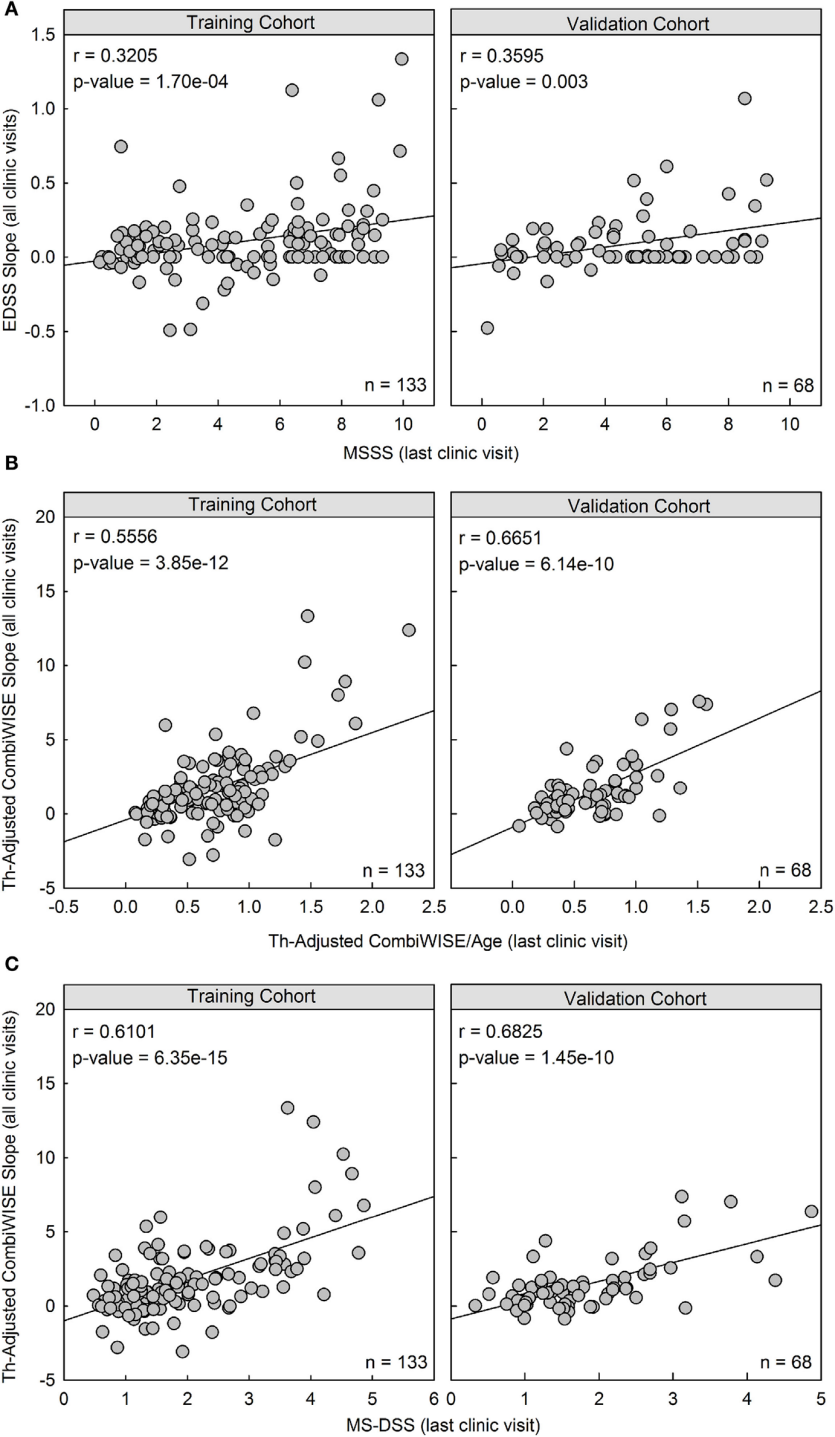
Therapy-adjusted CombiWISE/age and MS disease severity scale (MS-DSS) (at last clinic visit) are strong predictors of prior disability measured with therapy-adjusted CombiWISE slopes. **(A)** Cross-sectional MS Severity Score (MSSS) measured at the last clinic visit moderately correlates with longitudinal Expanded Disability Status Scale (EDSS) regression slopes. **(B,C)** The correlation markedly improves when using both cross-sectional therapy-adjusted CombiWISE/age **(B)** and subsequent MS-DSS **(C)** to predict therapy-adjusted CombiWISE regression slopes. Results are shown separately for the training cohort (Left panels; *n* = 133) and validation cohort (Right panels; *n* = 68). MSSS and CombiWISE values were calculated using published formulas (3, 10), and subsequent EDSS and therapy-adjusted CombiWISE regression slopes were derived using least squares fitting for patients with an average follow-up of 4.49 years.

While predicting future MS course is important in clinical applications, reliable measurement of MS severity is absolutely required in scientific efforts to identify biological processes and genetic modifiers that determine the speed of CNS tissue destruction. Our primary goal in MS-DSS development was to facilitate such studies. However, most historical cohorts with stored biological samples lack the data necessary for calculating CombiWISE or MS-DSS. It is likely that a significant portion of patients from these historical cohorts can be called back for a comprehensive follow-up visit that would allow collection of all data necessary for computing therapy-adjusted CombiWISE/age. Therefore, we asked how well both therapy-adjusted CombiWISE/age and MS-DSS computed at the *last* clinic visit correlated with prospectively acquired clinical data and whether both measures outperformed MSSS in this regard (Figure 5).

In both the training cohort and the validation cohort, MSSS measured at the last clinic visit had a moderate and significant correlation with previous disease progression measured by EDSS (Training: *r* = 0.3205, *p* = 1.70e−04; Validation: *r* = 0.3595, *p* = 0.003; Figure 5A). However, the correlation between therapy-adjusted CombiWISE/age and therapy-adjusted CombiWISE slopes markedly improved (Training: *r* = 0.5556, *p* = 3.85e−12; Validation: *r* = 0.6651, *p* = 6.14e−10; Figure 5B). MS-DSS also correlated strongly with therapy-adjusted CombiWISE slopes (Training: *r* = 0.6101, *p* = 6.35e−15; Validation: *r* = 0.6825, *p* = 1.45e−10; Figure 5C).

### Interactive Web Application for Computing MS Scales

**Figure 6:**
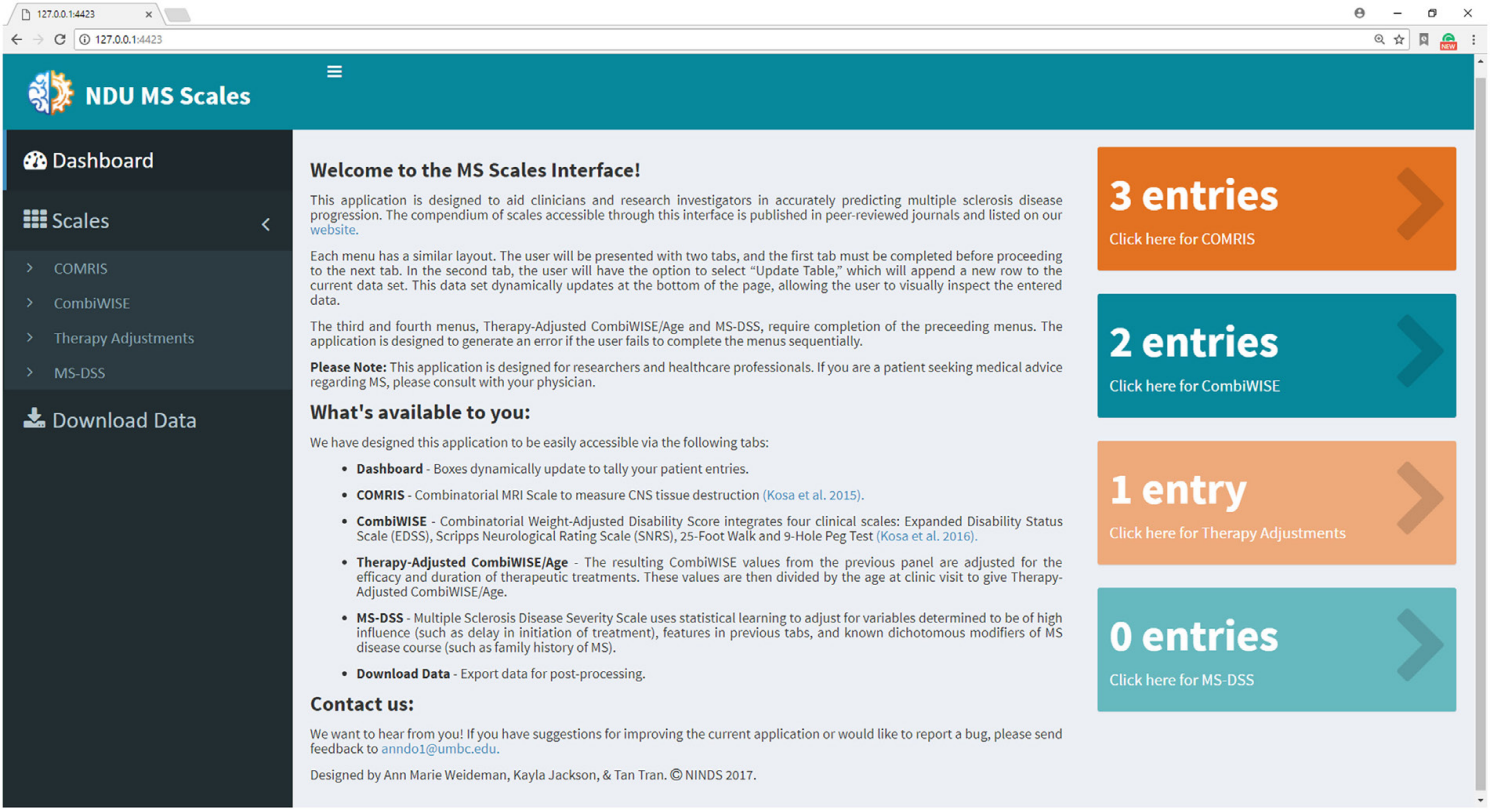
Dashboard (home page) within Shiny web application. Dashboard page introduces users to the application and allows them to monitor the status of each menu by dynamically updating the number of patient entries. Users navigate between four submenus (COMRIS (11), CombiWISE (10), therapy-adjusted CombiWISE/age, and MS-DSS). Within each submenu, the user is asked to input both continuous and categorical patient-specific features using the sliding scales, free text, and drop-down options. As the user enters data within each tab, results from intermediate computations are displayed in tabular form, enabling the user to parse through the dataset and examine output. This user-friendly interface was developed using the Shiny package in R (20).

**Figure 7:**
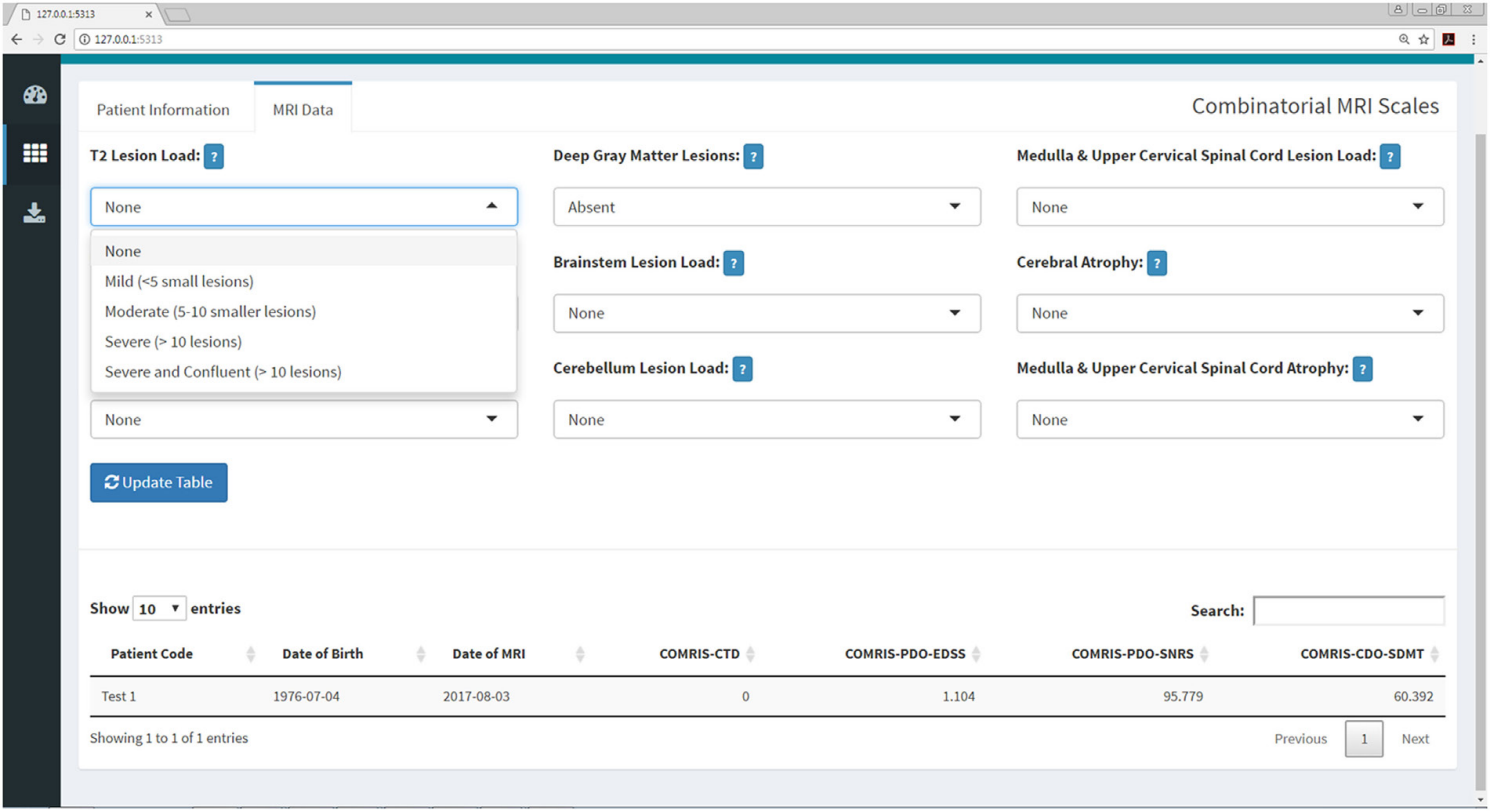
COMRIS submenu within the Shiny web application. This submenu allows the user to calculate four COMRIS values (COMRIS-CTD, COMRIS-PDO-EDSS, COMRIS-PDO-SNRS, and COMRIS-CDO-SDMT) by semi-quantitative grading of 9 MRI categories from 1.5T or 3T MRI. Each drop-down menu allows the user to select the lesion load or level of atrophy necessary to grade the MRI. Users who are unfamiliar with COMRIS can click the help menu beside each drop down for a brief description of the grading procedure and a link to the published article (11).

**Figure 8:**
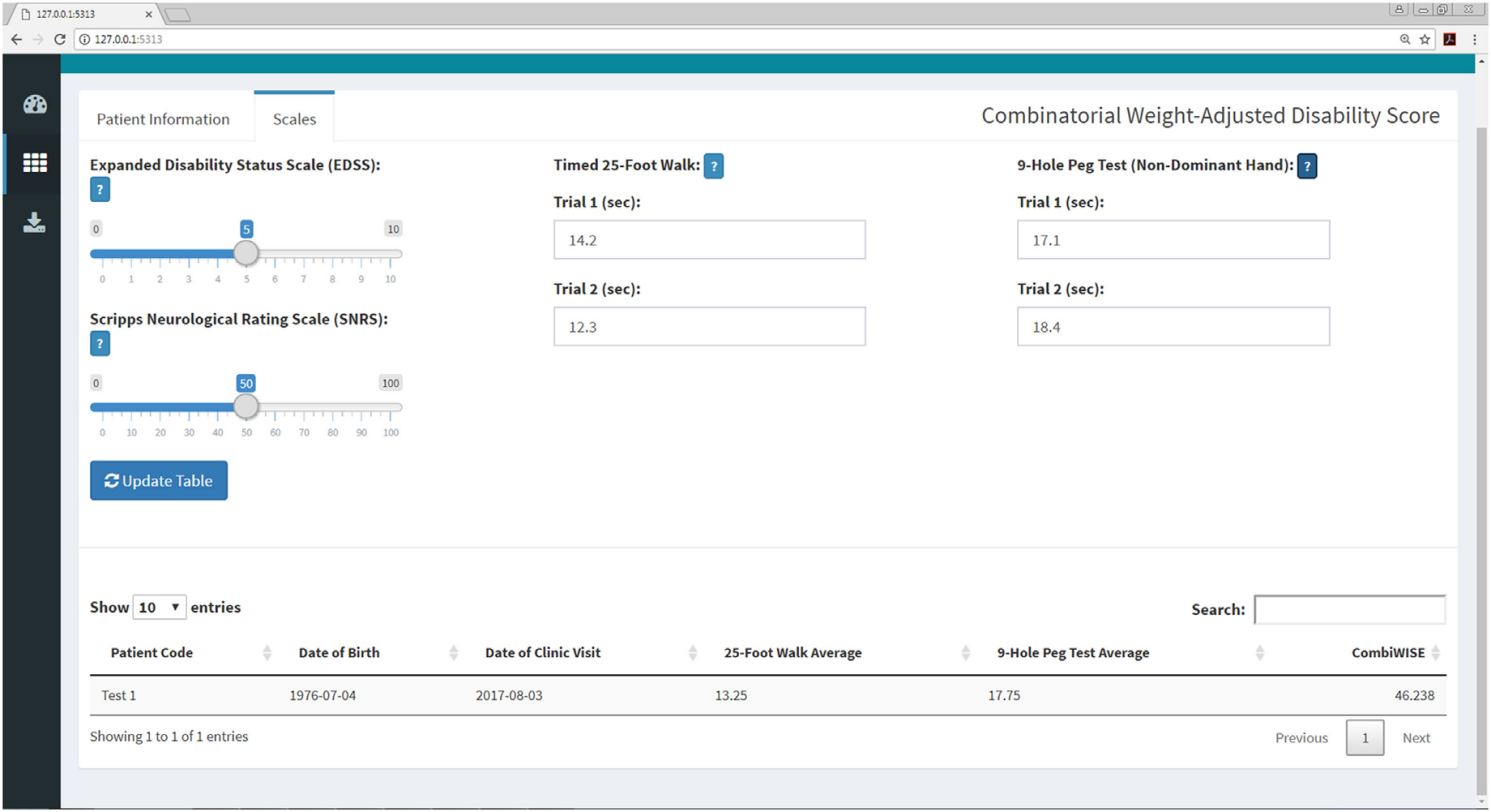
CombiWISE submenu within the Shiny web application. This submenu allows the user to input values for four scales [Expanded Disability Status Scale (EDSS), SNRS, Timed 25-Foot Walk (T25FW), and 9-Hole Peg Test (9HPT)] to compute CombiWISE (10). The values for EDSS (0, 1–10, incrementing by 0.5 where 0 is no disability and 10 is deceased) and SNRS (0–100, incrementing by 1.0 where 100 is no disability and 0 is deceased) are entered *via* sliding scales, and the T25FW and 9HPT are entered as free text. The T25FW and 9HPT have maximum time limits of 180 s (3 min) and 300 s (5 min), respectively. If these limits are surpassed, the application will prompt the user to round the value to the testing limit. The application controls for the entry of nonsensical data (e.g., 0 s trial) or non-numeric data (e.g., fifteen rather than 15) as demonstrated in Figure 11.

**Figure 9:**
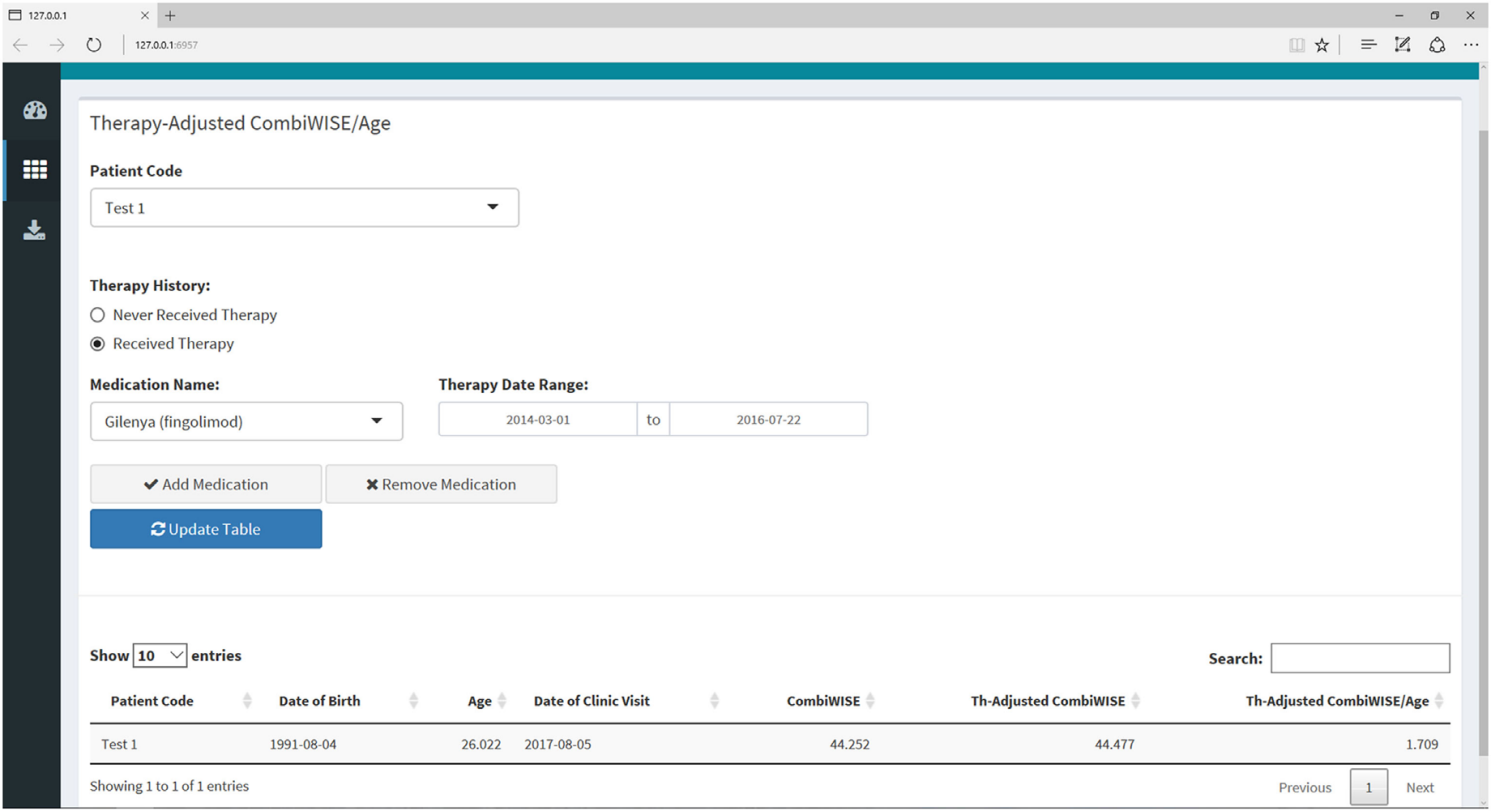
Therapy-adjusted CombiWISE/age submenu within the Shiny web application. This submenu allows the user to enter the patient’s medication history as needed for calculation of MS disease severity scale (MS-DSS) in the final tab. The medication history can be edited by adding or removing medications or amending the dates of administration. The list of medications only includes those that fulfill criteria for low- or high-efficacy as detailed in a recent meta-analysis of randomized, blinded clinical trials of immunomodulatory therapies in MS (14). If a patient has received a drug that is not listed in the drop-down menu, the program will treat this medication as zero efficacy in the calculation of MS-DSS.

**Figure 10:**
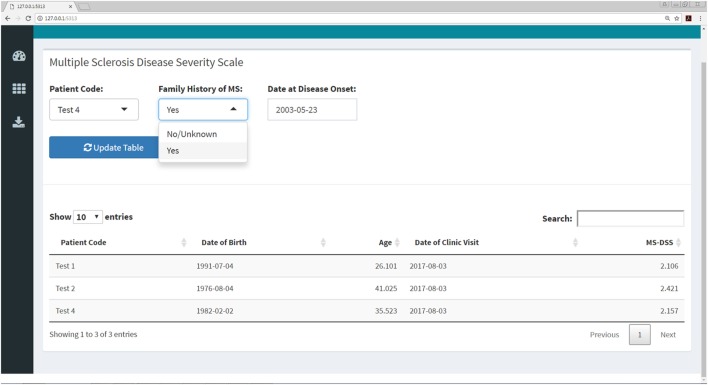
MS disease severity scale (MS-DSS) submenu within Shiny web application. This submenu allows the user to compute MS-DSS by entering information regarding family history and age at disease onset. All other features required for MS-DSS are computed on the server side of the application using the previously entered demographic information, medication history, auxiliary and neurological scales, and MRI grades. If the user does not have MRI grades within the past year, the application will treat this information as missing and use other available features to compute MS-DSS.

**Figure 11:**
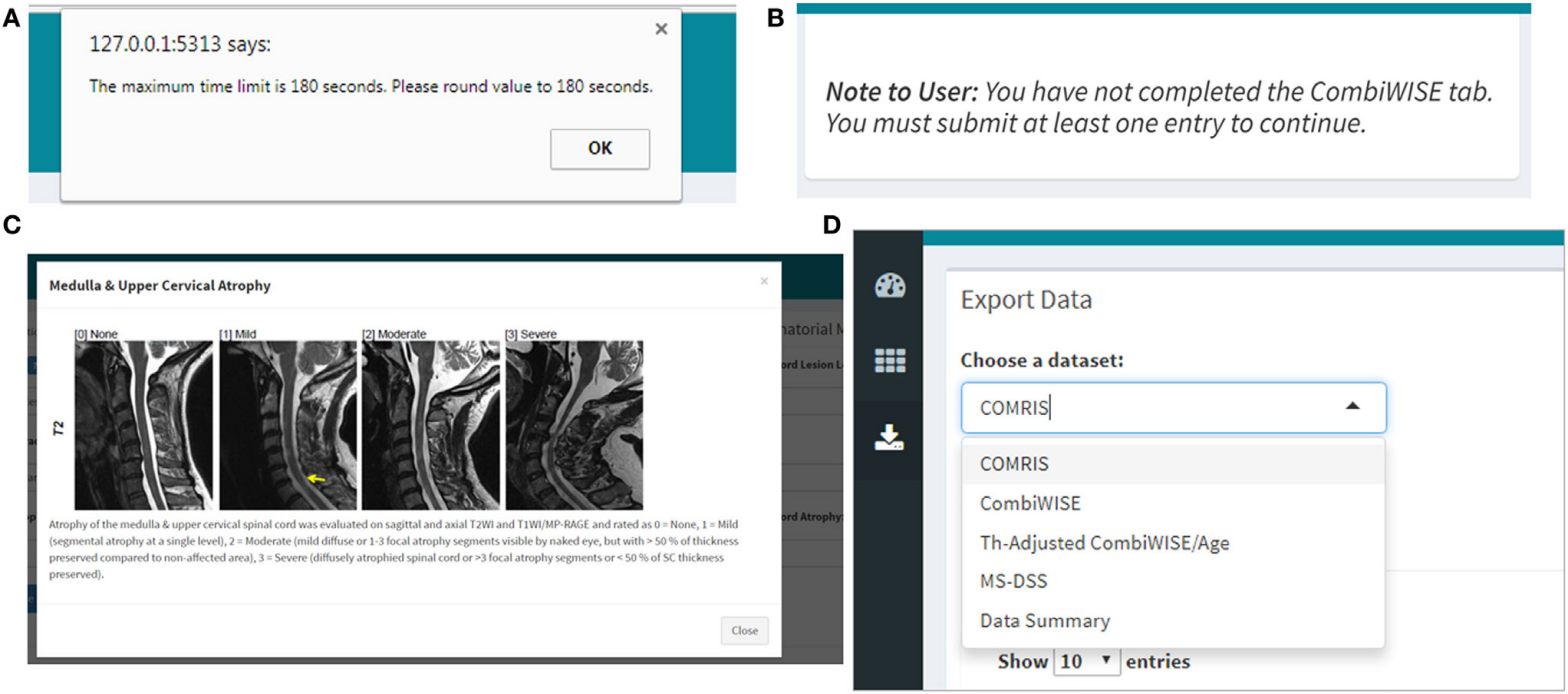
Quality control, help menus, and export within the Shiny web application. **(A)** If the user enters data that are non-numeric (e.g., ten instead of 10) or outside of the scope of the calculation (e.g., 0 second trial), the application will automatically generate a warning message with specifics regarding the error and directions on how to proceed. **(B)** The user must complete the COMRIS (11) and CombiWISE (10) tabs before proceeding to the therapy-adjusted CombiWISE/age or MS-DSS tabs. A notice is generated if the user attempts to access these tabs without having at least one entry within the preceding tabs. **(C)** A pop-up help menu is available beside each drop down or text box to assist users who are unfamiliar with the scales. Each help menu displays an image which is an active link to the published article from which the scale was derived. **(D)** When the desired computations are complete, the users can access the “Download Data” menu to export select or combined data sets.

In previous manuscripts (10, 11), we provided formulas and datasheets for computing COMRIS-CTD and CombiWISE; however, GBM-based modeling is not amenable for this type of public distribution. Therefore, we developed a user-friendly web interface using the Shiny package in R (20). Screenshots of the application (Figures 6–11) demonstrate the main features of this interface, which allows any user to readily compute all values from current and previously developed scales, including CombiWISE (10), COMRIS-CTD (11) and now MS-DSS, without any mathematical knowledge or programming skills. The user can access tabs and monitor data updates through the Dashboard page (Figure 6), and, within each submenu (Figures 7–10), the application displays the results in tabular form, allowing the user to parse through the dataset and examine output. If the user attempts to enter non-numeric data (e.g., fifteen instead of 15) or nonsensical data (e.g., 0 second trial), the application generates a gentle warning message (Figure 11A), and if the user attempts to proceed to the adjusted CombiWISE/age or MS-DSS tabs without completing the preceding tabs, the application will generate a notice to redirect the user (Figure 11B). A pop-up help menu is available beside each scale computation, and (where applicable) the images within each help menu are active links to the published article for that scale (Figure 11C). When the necessary computations are complete, the user can export individual datasets or an entire data summary as a CSV file (Figure 11D). The MS-DSS application can accessed at https://bielekovalab.shinyapps.io/msdss/.

## Discussion

Statistical learning is an indispensable technique for analyzing clinical data if the collected data has sufficient power to reliably measure existing relationships. Statistical power depends on the effect sizes of the processes we desire to investigate, the quantity of available measurements, and the precision of these measurements. Effect size is biologically determined, and, thus, cannot be modified by experimental design. Effect sizes tend to be small when considering individual elements, such as genes, proteins, or metabolites, in human polygenic diseases (21). Increasing the quantity of measurements (e.g., number of patients, frequency of visits, and length of follow-up in longitudinal studies) can compensate for small effect sizes, but cannot (or only to a limited degree) compensate for measurement imprecision. Despite this fact, greater emphasis is typically placed on increasing sample size than on optimizing measurement precision. This strategy not only inflates costs but may not yield the desired knowledge. If the measurement error exceeds the effect size, distinguishing biological relationships from measurement noise can become impossible, irrespective of sample size. Thus, it is essential that we understand the source and extent of measurement noise and use this knowledge to meaningfully improve measurement tools and thus decrease necessary sample sizes. This strategy is a win–win situation both for science and clinical care: decreasing sample size will promote more cost-effective science, while increasing precision will shift prediction accuracy from a group-level to a patient-level.

Our finding, that EDSS lacks the sensitivity to reliably measure disease progression in intervals shorter than 10 years, agrees with a recent observational study of 17,365 MS patients that measured a median annualized EDSS change of 0.1 (22), which corresponds to a one-point increase in EDSS occurring, on average, every 10 years. Consequently, the observed inability of the past disease course reflected by MSSS or ARMSS to predict future EDSS progression in longitudinal studies shorter than 10 years is, to a large degree, an artifact of the insensitive measurement of this discrete scale. When disability progression rates were measured in the same patients by the continuous CombiWISE scale, we observed a statistically significant, albeit weak, correlation between MSSS (or ARMSS) and CombiWISE slopes. Furthermore, in measurements of disability accumulation, it was possible to explain, on average, a larger portion of CombiWISE change over time than for EDSS, providing unequivocal evidence for the superiority of CombiWISE.

Another source of imprecision comes from assessing DD. There is a wealth of knowledge regarding subclinical formation of MS lesions (e.g., in patients with radiologically isolated syndrome) and equally subclinical loss of neurological functions that a patient may not perceive as disability, depending on his/her physical and cognitive activity levels. This makes exact determination of MS onset impossible. On the other hand, large observational studies demonstrated that EDSS disability milestones are accumulated generally as a function of age rather than DD (6, 23). This has been interpreted as evidence that accelerated aging, rather than MS-specific processes, such as inflammation, drive disability accumulation in MS. An alternative interpretation, supported by migration studies (24), genetic data (2), biomarker studies (18), and this paper, is that all MS subtypes represent a single disease process. This process is initiated, *on average*, at the same age in late childhood/early adulthood, but with an individually variable duration of the subclinical stage. Thus, adjusting disability measurements for age, rather than DD, lowers the error rate and strengthens the predictive power of MS-DSS. Even for the ARMSS/MSSS comparison, we observed slightly better performance of ARMSS (which uses age) compared to MSSS (which uses DD).

Another apparent source of error stems from the disregard of administered therapies in MSSS computation. We utilized a regression model from a recent meta-analysis of randomized, blinded clinical trials of MS DMTs (14) to adjust disability values for prior therapy. This adjustment removed the confounding effects of therapy to provide a better measure of patient progression rates. The improvement in correlations between measured CombiWISE/age and measured CombiWISE slopes versus therapy-adjusted CombiWISE/age and therapy-adjusted CombiWISE slopes were only modest. However, when testing the GBM model with unadjusted versus therapy-adjusted features, the therapy adjustments resulted in a more robust model with heightened predictive power. We believe that therapy adjustment represents a significant advance to the MS-DSS model, but its true value may have been underestimated in the current study. Our natural history protocol focuses on diagnostic work-up of neuroimmunological disorders, so most relapsing-remitting multiple sclerosis patients were untreated at their first clinic visit. The second large group of participants in our natural history protocol are patients with progressive MS (especially untreated primary progressive multiple sclerosis patients) who were screened into our progressive MS trials. Thus, this large proportion of patients was also mostly untreated. Consequently, a relatively small portion of patients underwent adjustments for past treatment, and, due to the advanced age of the progressive MS cohort, these patients also had no adjustment or only minor adjustment for therapy received during the follow-up period. Therapy adjustment also allows clinicians (and patients) to use the MS-DSS web app to predict average effect(s) on disability progression for low- versus high-efficacy therapy in comparison to remaining untreated.

The final step in the refinement of MS-DSS was the application of unbiased statistical learning. We used features that were previously shown to influence MS severity, such as gender and smoking. We also tested new features that, based on clinical knowledge, we considered likely to influence the rate of MS progression. Our hypothesis, that an increase in CNS tissue destruction (reflected by COMRIS-CTD and CombiWISE) will hasten disease progression because it limits functional recovery (i.e., formation of new synapses/circuits), was strongly supported by the GBM relative influence metrics, which selected these features above currently known modifiers of MS course. Similarly, delayed initiation of treatments was the fourth most important feature in GBM model, validating our hypothesis that such delay promotes establishment of compartmentalized inflammation and enabling us to identify patients with inadequate efficacy of administered DMTs. Analogously, the difference between therapy-adjusted CombiWISE and measured CombiWISE may reflect the beneficial effect of past treatments and, therefore, identify types of MS that are amenable to immunomodulation. It is important to recognize that none of the new features had better power in predicting future disability progression than therapy-adjusted CombiWISE/age. Thus, the new features are important modifiers that strengthen the predictive power of therapy-adjusted CombiWISE/age.

We acknowledge the following limitations: (1) our cohort was small in comparison to previous studies of MS severity models (3, 5) and the length of follow-up was limited. To mitigate this effect and to assure general reproducibility, we employed an independent validation cohort, not utilized for model development. The level of statistical significance in this comparatively smaller cohort indicated that, thanks to the high sensitivity of CombiWISE, we were not underpowered to detect meaningful relationships. (2) We employed a careful partitioning strategy described in the methods to balance the distribution of variables that we hypothesized *a priori* would be important for predicting disease progression. This ensured that the dataset used for model construction (and prediction of disease progression) was representative of a broad range of subjects. We acknowledge that this represents only one of several possible patient partitions. To estimate the variability in the model construction using entirely random training/validation splits, we considered 5,000 random partitions of the training and validation datasets. For each partition, a GBM model was constructed using the training dataset, predictions were made on the validation dataset, and the correlation between adjusted CombiWISE slopes and model predictions were recorded. The resulting distribution of the validation correlations from the retrained GBM models demonstrated that our obtained result falls in the top 97.1% of the results of such random training/validation cohort partitions. The mean validation correlation in these randomly re-constructed training/validation splits was 0.37 ± 0.10, which is still substantially better than performance of MSSS or ARMSS and marginally better than therapy-adjusted CombiWISE/age. (3) We made multiple assumptions in model construction that either generalize or oversimplify complex processes that contribute to disease progression. Nevertheless, the congruency between modeling and validation cohorts, and the statistical significance of individual comparisons with MSSS and ARMSS, provides assurance that the assumptions introduced in MS-DSS are consistent with the biological behavior of the MS disease process. (4) Because our dataset lacks a pediatric population, MS-DSS will not reliably measure MS progression in patients below age 21. Instead, we hope that pediatric MS centers will adopt our methodology to devise predictive models for pediatric patients.

**Figure 12:**
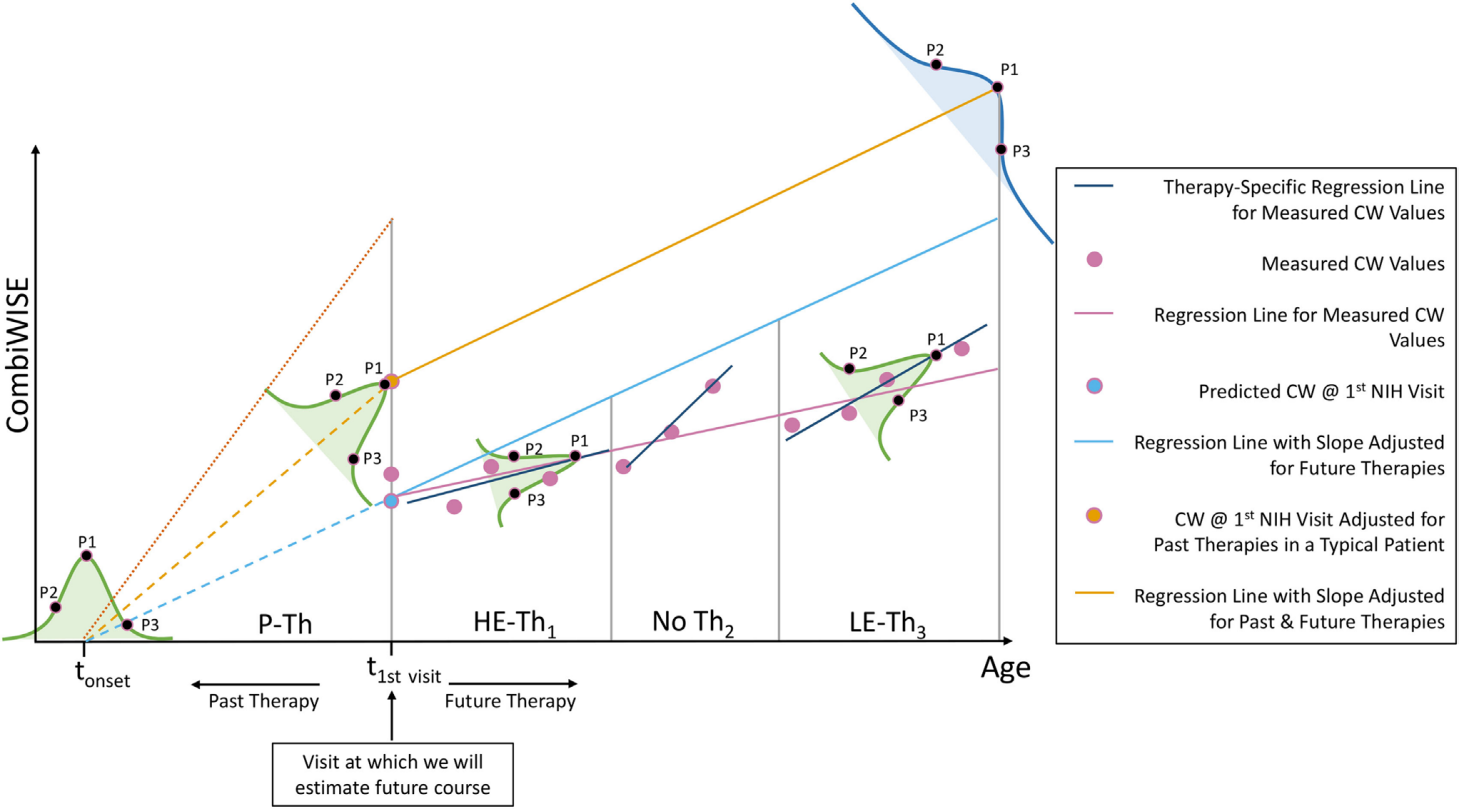
Explanation of additional sources of variation in the MS disease severity scale (MS-DSS) model. Every adjustment we made to the MS-DSS model is based on average values from large populations. Underlying each of these statistical averages lies a distribution (e.g., Gaussian) represented by the bell-shaped curves. The precise location of a patient within these distributions depends on patient-specific genetic, biological, and environmental factors. We will describe three types of theoretical patients that may be highly informative, even though each represents a rare variant. Assume that a patient P1 has precisely average behavior on all distributions. S/he will have an average age of multiple sclerosis (MS) onset (*t*_onset_), average response to high-efficacy therapy (HE-Th_1_), and average response to low-efficacy therapy (LE-Th_3_). Furthermore, assume that patient P2 has unusually severe MS behavior, and patient P3 has unusually mild MS behavior. When compared to the average patient (P1), P2 experienced MS onset at an early age and did not respond well to high- or low-efficacy therapy; whereas, P3 experienced MS onset at a later age and responded well to high- or low-efficacy therapy. Finally, all patients will end within the blue distribution, which comprises all variation demonstrated by the green distributions and is, therefore, the broadest. Within this distribution, P1 will fall precisely at the MS-DSS predicted average (peak); whereas, P2 will have higher than average MS-DSS predicted disability, and P3 will have lower than average MS-DSS predicted disability. The resulting behavior of P2 and P3 within each of these distributions can be attributed to a variety of patient-specific factors that are not included in MS-DSS model, such as genetic predisposition to more aggressive (P2) or milder (P3) MS, and environmental factors such as EBV infection, smoke exposure, low vitamin D, obesity, lack of exercise, etc. The MS-DSS model cannot make more accurate individual predictions until we understand all genetic, biological, and environmental modifiers and include them in the model.

We also acknowledge that the current MS-DSS model might be further improved by adding biological data. As of this study, all models of MS severity, including MSSS, ARMSS, and MS-DSS, are based on the behavior of an *average* MS patient. However, as depicted in the Figure 12, behind every statistical *average* lies a distribution (e.g., Gaussian) from which this *average* is derived. The exact location a patient falls on this distribution is determined by patient-specific biological or environmental factors. The wider the spread in this distribution, the more imprecise the patient-specific predictions are when derived from a model that captures only group behaviors. Therefore, it is not surprising that, without inputting patient-specific data (e.g., genotypes, protein, and metabolic biomarkers), the prognostic power of MS-DSS can be only moderate. Based on the results of this study, we expect that inclusion of patient-specific biological features will serve to further strengthen the predictive power of MS-DSS. However, identification and validation of biological features that predict response to treatment or progression rates is missing precisely because we have, thus far, lacked a sensitive MS severity scale necessary for generating this knowledge in reasonably sized cohorts.

We have also demonstrated that therapy-adjusted CombiWISE/age can be applied “backwards” without any loss of sensitivity by collecting CombiWISE measurements at the post-treatment stage and adjusting these measurements for previous therapeutic history. This allows utilization of existing MS sample repositories given that the patients can be called back and re-examined. For the patients who cannot be re-examined, there are two additional solutions if only SNRS is missing: (1) SNRS can be generated retrospectively from a documented neurological exam, or (2) if a detailed neurological exam is not available, SNRS can be successfully approximated from any 1.5T or 3T brain and upper spinal cord MRI using COMRIS-based models (11).

The final limitation of MS-DSS, which may hinder its widespread acceptance, is its complexity. The user-friendly web application that we have developed allows any user, devoid of knowledge in mathematics, statistics or programming, to derive all scales that we have developed [COMRIS-CTD (11), CombiWISE (10) and now MS-DSS]. In addition, the application allows clinicians (and patients) to design potential therapeutic regimens and then use these data to extrapolate average disability progression in an untreated versus treated state. For COMRIS-CTD, the application utilizes widgets and help menus to guide the clinician through semi-quantitative MRI rating, a process that can easily be performed by any MS-trained clinician. To compute CombiWISE, the user must input four different scales (EDSS, SNRS, T25FW, and 9HPT). The aforementioned tabs must be completed to proceed to calculation of MS-DSS. If the user fails to complete these tabs, the program will block progress through the application until the necessary information is entered, as both scales are required for computation of MS-DSS. If the user enters MRI data that are within a year of the visit date, the application will use this COMRIS data within the MS-DSS model. Otherwise, the information will be treated as missing data and the computation will proceed using all other available features. This process may seem overwhelming to clinicians or investigators who are accustomed to only collecting EDSS. Unfortunately, precision cannot be achieved without collecting *multiple* features that together capture the complexity of the disease process. EDSS alone cannot be the sole basis of a highly reproducible model of MS severity, unless the average follow-up spans decades. Unfortunately, with the current motility of the world-wide human populations, the attrition rates of such long-term cohorts are unacceptably high. Also, the cost and practicalities of maintaining such large, long-term cohorts are exceedingly demanding. Instead, we can achieve progressively higher levels of precision if we collect data capable of explaining residual variance. Only then can we hope to achieve precision that will allow us to learn from smaller cohorts and apply this knowledge to individual patients. Precision medicine demands precise/complex measurements.

In conclusion, MS-DSS has the potential to correctly identify relationships between MS severity and genes, proteins, or metabolites in significantly smaller cohorts than required for MSSS or its ARMSS analog. While MS-DSS can aid decisions about personalized treatments in its current form, future enhancements of the model with patient-specific biological data will unquestionably strengthen its individualized predictive power.

## Ethics Statement

This study was carried out in accordance with the recommendations of Declaration of Helsinki with written informed consent from all subjects. The protocol was approved by the NIH CNS institutional Review Board (IRB).

## Author Contributions

BB was responsible for original study concept, design, and supervision. BB and AW were responsible for acquisition of clinical data, and PK was responsible for data export. PK, MK, and BB were responsible for design of COMRIS-CTD (MRI scale) utilized as a feature in the model. AMW, CB, MT-M, MG, KJO, and BB were responsible for model design and statistical analyses. AMW, KJA, and TT developed and tested the web application. AMW, CB, MG, and BB were responsible for drafting the text. All authors contributed to revision and approval of the final manuscript.

## Conflict of Interest Statement

The authors declare that the research was conducted in the absence of any commercial or financial relationships that could be construed as a potential conflict of interest.

## Abbreviations

ARMSS, age related multiple sclerosis severity score; CDP, confirmed disability progression; CNS, central nervous system; CombiWISE, combinatorial weight-adjusted disability score; COMRIS-CTD, combinatorial MRI score of CNS tissue destruction; DD, disease duration; DMT, disease modifying therapy; EDSS, expanded disability status scale; GBM, gradient boosting machine; MRI, magnetic resonance imaging; MS-DSS, MS disease severity scale; MSSS, MS severity score; NIH, National Institutes of Health; 9HPT, 9-hole peg test; PPMS, primary progressive multiple sclerosis; QC, quality control; *r*, Pearson correlation coefficient; *R*^2^, coefficient of determination; RRMS, relapsing-remitting multiple sclerosis; SNRS, Scripps neurological rating scale; T25FW, timed 25-foot walk.
