# Supplementary material for: New Multiple Sclerosis Disease Severity Scale Predicts Future Accumulation of Disability
Source: Front Neurol. 2017 Nov 10;8:598. doi: 10.3389/fneur.2017.00598 (PMC5686060; doi:10.3389/fneur.2017.00598)
Supplement: Supplementary file 1 [file Presentation_1.PDF]

Supplementary Material

Script S1: R code for modeling therapy adjustments to CombiWISE values.

```
#---------------------------------------------------------------------------------------------------------
#PURPOSE: Therapy adjustments for multiple sclerosis disease severity scale (MS-DSS)
#INPUT: Demographics and disability measures of 201 patient cohort
#OUTPUT: Raw and adjusted CombiWISE values needed for gradient boosting machine modeling of MS-DSS
#---------------------------------------------------------------------------------------------------------
#AUTHOR:
#Ann Marie Weideman, annmarie.weideman@nih.gov
#DATE: 5/26/17
#UPDATED: 9/29/2107
#---------------------------------------------------------------------------------------------------------

#only need to install packages once, then comment out
#install.packages("dplyr")
#install.packages("R.utils")
#install.packages("readxl")
#install.packages("data.table")
#install.packages("plyr")
#install.packages("Matrix")
#install.packages("stats")
#install.packages("cocor")
#install.packages("L1pack")
#install.packages("PairedData")
#install.packages("ms.sev")

#open libraries
library(dplyr)
library(tidyr)
library(R.utils)
library(readxl)
library(data.table)
library(plyr)
library(Matrix)
library(stats)
library(L1pack)
```

```r
library(PairedData)
library(ms.sev)

#------------------------------------------------------------------------------------------------------
#Library of functions
#------------------------------------------------------------------------------------------------------


#Function to read in multiple excel sheets
read_excel_allsheets <- function(filename) {
  sheets <- readxl::excel_sheets(filename)
  x <-    lapply(sheets, function(X) readxl::read_excel(filename, sheet = X))
  names(x) <- sheets
  x
}

#function to compute efficacy of a low efficacy drug
#Input: Treatment duration
#Ouput: Therapy efficacy
fun.lowEff <- function(x) {
  eff <-  -1.50309106452*mean(c(olaps.min[x],olaps.max[x]))+83.70699018595
  return(eff)
}

#function to compute efficacy of a high efficacy drug
#Input: Treatment duration
#Ouput: Therapy efficacy
fun.highEff <- function(x) {
  eff <-  -4.34492881207*mean(c(olaps.min[x],olaps.max[x]))+206.39259418775
  return(eff)
}

#function to find matches
fun.matches<-function(x,y) {which(x==y)}

#------------------------------------------------------------------------------------------------------
#Read and clean dataset
#------------------------------------------------------------------------------------------------------

#read in three sheets of xlsx file
mysheets <- read_excel_allsheets("Copy of Longitudinal data for MSDSS_minusPeds_06092017.xlsx")
```

```
sheet1.df<-mysheets$CombiWISE
sheet2<-mysheets$DMT
sheetCOMRIS<-mysheets$COMRIS_CTD

#partition data by NIB number (patient ID)
sheet1.splitData<-split(sheet1.df,sheet1.df[1])
sheetCOMRIS.splitData<-split(sheetCOMRIS,sheetCOMRIS[1])

#add COMRIS_CTD data to sheet1
for (i in 1:length(sheet1.splitData)){
  sheet1.splitData[[i]]$COMRIS.CTD.firstvisit<-sheet1.splitData[[i]]$COMRIS_first
  sheet1.splitData[[i]]$COMRIS.CTD.lastvisit<-sheet1.splitData[[i]]$COMRIS_last
}

#unsplit lists
sheet1.df<-do.call("rbind", sheet1.splitData)
sheetCOMRIS<-do.call("rbind", sheetCOMRIS.splitData)

#set aside all values present in sheet1 that are not in sheet2 (i.e. patients with clinical visits and no
#DMT)
i.omit<-which(is.na(match(sheet1.df$PatientCode,sheet2$PatientCode)))
sheet1.omit<-sheet1.df[i.omit,]
sheet1.omit.splitData<-split(sheet1.omit,sheet1.omit[1])

#append demographics and disease measures to sheet1.omit.splitdata (data set for patients who did not
#receive therapy)
for (i in 1:length(sheet1.omit.splitData)){
  sheet1.omit.splitData[[i]]$CombiWISE.predicted.firstvisit<-
rep(sheet1.omit.splitData[[i]]$CombiWISE[1],nrow(sheet1.omit.splitData[[i]]))
  sheet1.omit.splitData[[i]]$CombiWISE.adjusted.firstvisit<-
rep(sheet1.omit.splitData[[i]]$CombiWISE[1],nrow(sheet1.omit.splitData[[i]]))
  sheet1.omit.splitData[[i]]$CombiWISE.predicted.lastvisit<-
rep(sheet1.omit.splitData[[i]]$CombiWISE[nrow(sheet1.omit.splitData[[i]])],nrow(sheet1.omit.splitData[[i]])
)
  sheet1.omit.splitData[[i]]$CombiWISE.adjusted.lastvisit<-
rep(sheet1.omit.splitData[[i]]$CombiWISE[nrow(sheet1.omit.splitData[[i]])],nrow(sheet1.omit.splitData[[i]])
)
  sheet1.omit.splitData[[i]]$Time2DMT.firstvisit<-
c(sheet1.omit.splitData[[i]]$DiseaseDuration[1],rep("",nrow(sheet1.omit.splitData[[i]])-1))
```

```
  sheet1.omit.splitData[[i]]$Time2DMT.lastvisit<-
c(sheet1.omit.splitData[[i]]$DiseaseDuration[(nrow(sheet1.omit.splitData[[i]]))],rep("",nrow(sheet1.omit.sp
litData[[i]])-1))
  sheet1.omit.splitData[[i]]$TxUnder6.firstvisit<-rep(0,nrow(sheet1.omit.splitData[[i]]))
  sheet1.omit.splitData[[i]]$TxUnder6.lastvisit<-rep(0,nrow(sheet1.omit.splitData[[i]]))
  sheet1.omit.splitData[[i]]$Gender<-
rep(sheet1.omit.splitData[[i]]$Gender[1],nrow(sheet1.omit.splitData[[i]]))
  sheet1.omit.splitData[[i]]$fhxMS<-
rep(sheet1.omit.splitData[[i]]$fhxMS[1],nrow(sheet1.omit.splitData[[i]]))
  sheet1.omit.splitData[[i]]$Smoke<-
rep(sheet1.omit.splitData[[i]]$Smoke[1],nrow(sheet1.omit.splitData[[i]]))
  sheet1.omit.splitData[[i]]$Race<-rep(sheet1.omit.splitData[[i]]$Race[1],nrow(sheet1.omit.splitData[[i]]))
}

#remove omitted patients from sheet1
sheet1<-sheet1.df[-i.omit,]

#convert all strings to lowercase (to avoid case dependent computations)
sheet1 <- mutate_all(sheet1, funs(tolower))
sheet2 <- mutate_all(sheet2, funs(tolower))

#pre-specify format for numeric values
sheet1$Age<-as.numeric(sheet1$Age)
sheet1$CombiWISE<-as.numeric(sheet1$CombiWISE)
sheet1$EDSS<-as.numeric(sheet1$EDSS)
sheet1$MSSS<-as.numeric(sheet1$MSSS)
sheet1$DiseaseDuration<-as.numeric(sheet1$DiseaseDuration)
sheet2$AgeInitiation<-as.numeric(sheet2$AgeInitiation)
sheet2$AgeTermination<-as.numeric(sheet2$AgeTermination)

#partition data by NIB number
sheet1.splitData<-split(sheet1,sheet1[1])
sheet2.splitData<-split(sheet2,sheet2[1])

#Count number of treatments that were taken for less than 6 months for each patient at the first clinic
#visit
for (i in 1:length(sheet1.splitData)){
  count<- 0
  for (j in 1:nrow(sheet2.splitData[[i]])){
    if ((sheet2.splitData[[i]]$AgeTermination[j]-sheet2.splitData[[i]]$AgeInitiation[j])<0.5 &
        sheet2.splitData[[i]]$AgeTermination[j]<=sheet1.splitData[[i]]$Age[1] &
```

```
        is.na(sheet2.splitData[[i]]$AgeTermination[j])==F){
      count<-count+1
    }
  }
  sheet1.splitData[[i]]$TxUnder6.firstvisit<-count
}

#Count number of treatments that were taken for less than 6 months for each patient at the last clinic
#visit
for (i in 1:length(sheet1.splitData)){
  count<- 0
  for (j in 1:nrow(sheet2.splitData[[i]])){
    if ((sheet2.splitData[[i]]$AgeTermination[j]-sheet2.splitData[[i]]$AgeInitiation[j])<0.5 &
        sheet2.splitData[[i]]$AgeTermination[j]<=last(sheet1.splitData[[i]]$Age) &
        is.na(sheet2.splitData[[i]]$AgeTermination[j])==F){
      count<-count+1
    }
  }
  sheet1.splitData[[i]]$TxUnder6.lastvisit<-count
}

#unsplit list
sheet2<-do.call("rbind", sheet2.splitData)

#for patients who continued therapy past their last visit, assume a termination date equal to the last
#clinical visit
i.NA<-which(sheet2$AgeTermination %in% NA)
NIB.NA<-sheet2$PatientCode[i.NA]
for (i in 1:length(NIB.NA)){sheet2$AgeTermination[i.NA[i]]<-
as.numeric(max(sheet1.splitData[[NIB.NA[i]]]$Age))}

#partition data by NIB number
sheet2.splitData<-split(sheet2,sheet2[1])

#order split lists by age and then unsplit
sheet1.splitData<-lapply(sheet1.splitData, function(x) x[order(x$Age),])
sheet2.splitData<-lapply(sheet2.splitData, function(x) x[order(x$AgeInitiation),])

#remove duplicate entries under "Age" for each NIB number
sheet1.splitData<-lapply(sheet1.splitData, function(x) x[!duplicated(x["Age"]), ])
```

```r
#find all treatments started before the last clinic date and ended after the last clinic date; set
#termination date to equal the last clinic date
for (i in 1:length(sheet2.splitData)){
  for (j in 1:nrow(sheet2.splitData[[i]])){
    if (max(sheet1.splitData[[i]]$Age)<sheet2.splitData[[i]]$AgeTermination[j]){
      sheet2.splitData[[i]]$AgeTermination[j]<-max(sheet1.splitData[[i]]$Age)}
  }
}

#initialize vector
vec.ind<-c()

#remove all treatments started after the last clinic date
for (i in 1:length(sheet2.splitData)){
  for (j in 1:nrow(sheet2.splitData[[i]])){
    if (max(sheet1.splitData[[i]]$Age)<sheet2.splitData[[i]]$AgeInitiation[j]){
      vec.ind<-c(vec.ind,j)}
  }
   if(length(vec.ind)>0){
   sheet2.splitData[[i]]<-sheet2.splitData[[i]][-vec.ind,]
   vec.ind<-c()
   }
}

#unsplit lists
sheet1<-do.call("rbind", sheet1.splitData)
sheet2<-do.call("rbind", sheet2.splitData)

#----------------------------------------------------------------------------------------------------
#Locate positions of low and high efficacy therapy and assign type:
#0 = untreated or unknown efficacy
#1 = low efficacy
#2 = high efficacy
#----------------------------------------------------------------------------------------------------

#create lists of low and high efficacy therapy
lowEff<-c("fingolimod","gilenya","interferon","avonex","betaseron","rebif","plegridy","teriflunomide",
"aubagio","glatiramer acetate","copaxone","dimethyl fumarate","tecfidera")

abv.lowEff<-c("fing","gile","inte","avon","beta","rebi","pleg","teri","auba","glat","copa","dime","tecf")
```

```
highEff<-
c("mitoxantrone","novantron","novantrone","natalizumab","tysabri","ocrelizumab","ocrevus","alemtuzumab","le
mtrada","daclizumab","zinbryta","zenapax","10-N-0125","99-N-0169","04-N-0019")

abv.highEff<-c("mito","nova","nata","tysa","ocre","alem","lemt","dacl","zinb","zena","0125","0169","0019")

#locate indices of low efficacy therapy
low.matches <- unique(grep(paste(abv.lowEff,collapse="|"), sheet2$Therapy, value=TRUE))
i.low<-tapply(seq_along(sheet2$Therapy), sheet2$Therapy, identity)[low.matches]
i.low<-as.numeric(unlist(i.low))
i.low<-sort(i.low)

#locate indices of high efficacy therapy
high.matches <- unique(grep(paste(abv.highEff,collapse="|"), sheet2$Therapy, value=TRUE))
i.high<-tapply(seq_along(sheet2$Therapy), sheet2$Therapy, identity)[high.matches]
i.high<-as.numeric(unlist(i.high))
i.high<-sort(i.high)

#create efficacy vector
sheet2$Efficacy<-numeric(length(sheet2$Therapy))
sheet2$Efficacy[i.low]<-1
sheet2$Efficacy[i.high]<-2 #any therapies classified as a low/high combo will then be classified as high

#partition data by NIB number
sheet1.splitData<-split(sheet1,sheet1[1])
sheet2.splitData<-split(sheet2,sheet2[1])
sheetCOMRIS.splitData<-split(sheetCOMRIS,sheetCOMRIS[1])

#-----------------------------------------------------------------------------------------------------
#Adjust end dates of high efficacy therapies
#-----------------------------------------------------------------------------------------------------
#Add one month to the termination date for all monoclonal antibodies and three months for
#Mitoxantrone/Novantrone in order to adjust for duration of the drug effect.

#Note: The first pass through adds one month to all high efficacy treatments; the second pass through adds
#two more months to adjust Mitoxantrone/Novantrone to three months.

sheet2$AgeTermination[i.high]<-sheet2$AgeTermination[i.high]+(1/12)

chemo.matches <- unique(grep(paste(c("mito","nova"),collapse="|"), sheet2$Therapy, value=TRUE))
i.chemo<-tapply(seq_along(sheet2$Therapy), sheet2$Therapy, identity)[chemo.matches]
```

```r
i.chemo<-as.numeric(unlist(i.chemo))
i.chemo<-sort(i.chemo)

sheet2$AgeTermination[i.chemo]<-sheet2$AgeTermination[i.chemo]+(1/6)

#resplit
sheet2.splitData<-split(sheet2,sheet2[1])

#-----------------------------------------------------------------------------------------------------
#Clean initiation age and termination age vectors
#-----------------------------------------------------------------------------------------------------

#intialize empty lists
sheet3.PatientCode <- c()
sheet3.AgeInitiation <- c()
sheet3.AgeTermination <- c()
sheet3.Efficacy<-c()
sheet3.Therapy<-c()

#loop through split data to create new initiation age and termination age vectors
for (i in 1:length(sheet2.splitData)){

  #create new vector of conjoined age initiation and termination values and sort data
  AgeInitiation<-sort(c(sheet2.splitData[[i]]$AgeInitiation,sheet2.splitData[[i]]$AgeTermination))

  #assign to vector
  sheet3.AgeTermination <- c(sheet3.AgeTermination,AgeInitiation)

  #delete final value on age initiation vector
  AgeInitiation<-AgeInitiation[-length(AgeInitiation)]

  #add untreated period from age zero to age of first therapy
  AgeInitiation <- c(0,AgeInitiation)

  #expand patient NIB vector to match length of current dataframe
  PatientCode<-rep.int(sheet2.splitData[[i]]$PatientCode[1], length(AgeInitiation))

  #assign to vectors
  sheet3.AgeInitiation <- c(sheet3.AgeInitiation,AgeInitiation)
  sheet3.PatientCode <- c(sheet3.PatientCode,PatientCode)
```

```
}

#append all vectors to dataframe
sheet3<- data.frame(
  PatientCode=sheet3.PatientCode,
  AgeInitiation=sheet3.AgeInitiation,
  AgeTermination=sheet3.AgeTermination,
  stringsAsFactors=FALSE)

#find indices where initiation and termination age match -> delete all matches
matches<-which(sheet3$AgeInitiation == sheet3$AgeTermination)
if (length(matches)>0){
  sheet3<-sheet3[-matches,]
} else{
  sheet3<-sheet3}

#partition new dataframe by NIB number
sheet3.splitData<-split(sheet3,sheet3[1])

#-----------------------------------------------------------------------------------------------------
#Construct vector containing max drug efficacy for each treated period
#-----------------------------------------------------------------------------------------------------

k<-1 #initialize counter

#intialize empty lists
sheet3b.PatientCode <- c()
sheet3b.AgeInitiation <- c()
sheet3b.AgeTermination <- c()
sheet3b.Efficacy<- c()
sheet3b.Therapy<-c()

#loop through split dataset by NIB number
for (i in 1:length(sheet2.splitData)){

  #loop through therapy start and end dates
  for (j in 1:length(sheet3.splitData[[i]]$AgeInitiation)){

    #find midpoint of each treatment period
    avg.therapy<-mean(c(sheet3.splitData[[i]]$AgeInitiation[j],sheet3.splitData[[i]]$AgeTermination[j]))
```

```
    #now search for all therapy that occurred within each treated period
    inds<-which(sheet2.splitData[[i]]$AgeInitiation <= avg.therapy & sheet2.splitData[[i]]$AgeTermination
>= avg.therapy)

    #find highest efficacy for that period
    #otherwise, leave as 0 for untreated
    if (length(inds)!=0) { sheet3b.Efficacy<-c(sheet3b.Efficacy,max(sheet2.splitData[[i]]$Efficacy[inds]))
    } else {sheet3b.Efficacy<-c(sheet3b.Efficacy,0)}

    k<-k+1 #update counter

  }

  #if therapy termination date occurs before last clinic date, then add untreated period to end of vector
  if (max(sheet3.splitData[[i]]$AgeTermination) < max(sheet1.splitData[[i]]$Age))
  {AgeInitiation<-c(sheet3.splitData[[i]]$AgeInitiation,max(sheet3.splitData[[i]]$AgeTermination))
  AgeTermination<-c(sheet3.splitData[[i]]$AgeTermination,max(sheet1.splitData[[i]]$Age))
  sheet3b.Efficacy<-c(sheet3b.Efficacy,0)

  #otherwise, duplicate vector from previous dataframe
  }else{AgeInitiation<-sheet3.splitData[[i]]$AgeInitiation
  AgeTermination<-sheet3.splitData[[i]]$AgeTermination}

  #assign to vectors
  PatientCode<-rep.int(sheet2.splitData[[i]]$PatientCode[1], length(AgeInitiation))
  sheet3b.AgeInitiation<-c(sheet3b.AgeInitiation,AgeInitiation)
  sheet3b.AgeTermination<-c(sheet3b.AgeTermination,AgeTermination)
  sheet3b.PatientCode <- c(sheet3b.PatientCode,PatientCode)
}

#create new dataframe
sheet3b<- data.frame(
  PatientCode=sheet3b.PatientCode,
  AgeInitiation=sheet3b.AgeInitiation,
  AgeTermination=sheet3b.AgeTermination,
  Efficacy = sheet3b.Efficacy,
  RawEfficacy=numeric(length(sheet3b.PatientCode)),
  AdjustedEfficacy=numeric(length(sheet3b.PatientCode)),
  stringsAsFactors=FALSE)

#partition new dataframe by NIB number
```

```r
sheet3b.splitData<-split(sheet3b,sheet3b[1])

#-----------------------------------------------------------------------------------------------------
#Create new age vector in order to align with clinic dates
#-----------------------------------------------------------------------------------------------------

#intialize empty lists
sheet4.PatientCode <- c()
sheet4.Age <- c()

#loop through split dataset by NIB number
for (i in 1:length(sheet2.splitData)){

  #find all ages that occur before the first clinic date
  Age<-sheet3b.splitData[[i]]$AgeInitiation[which(sheet3b.splitData[[i]]$AgeInitiation <
  min(sheet1.splitData[[i]]$Age))]

  #concatenate with visit ages from sheet1 to create a new vector of visit ages
  Age<-c(Age,sort(sheet1.splitData[[i]]$Age))
  sheet4.Age <- c(sheet4.Age,Age)

  #expand patient NIB vector to match length of current dataframe
  PatientCode<-rep.int(sheet2.splitData[[i]]$PatientCode[1], length(Age))
  sheet4.PatientCode <- c(sheet4.PatientCode,PatientCode)

  #append all vectors to dataframe
  sheet4<- data.frame(
    PatientCode=sheet4.PatientCode,
    AgeInitiation=sheet4.Age,
    stringsAsFactors=FALSE)

}

#rename columns in dataframe
names(sheet4)[names(sheet4)=="AgeInitiation"] <- "AgeatVisit"

#partition new dataframe by NIB number
sheet4.splitData<-split(sheet4,sheet4$PatientCode)

#remove duplicate entries under "AgeatVisit" for each NIB number
sheet4.splitData<-lapply(sheet4.splitData, function(x) x[!duplicated(x["AgeatVisit"]), ])
```

```
#unsplit list
sheet4<-do.call("rbind", sheet4.splitData)

#--------------------------------------------------------------------------------------------------------
#Create new vector with cumulative efficacy for each time period
#--------------------------------------------------------------------------------------------------------

#initialize empty lists
olaps.min<-c()
olaps.max<-c()
cum.eff<-c()
first.cum.eff<-c()
last.cum.eff<-c()
future.cum.eff<-c()
eff.cat<-c()

#loop through values in sheet3b dataset split by NIB number
for (i in 1:length(sheet3b.splitData)){

  #create dataframe of ranges between clinic visits
  AgeatVisit.n1<-sheet4.splitData[[i]]$AgeatVisit[-(nrow(sheet4.splitData[[i]]))]
  AgeatVisit.n2<-sheet4.splitData[[i]]$AgeatVisit[-1]
  VisitRanges<- data.frame(AgeatVisit.n1=AgeatVisit.n1,AgeatVisit.n2=AgeatVisit.n2, stringsAsFactors=FALSE)

  #create dataframe of therapy durations
  AgeInitiation<-sheet3b.splitData[[i]]$AgeInitiation
  AgeTermination<-sheet3b.splitData[[i]]$AgeTermination
  TherapyRanges<- data.frame(AgeInitiation=AgeInitiation,AgeTermination=AgeTermination,
  stringsAsFactors=FALSE)

  #key vectors to speed-up finding overlaps
  setkey(setDT(VisitRanges), AgeatVisit.n1, AgeatVisit.n2)
  setkey(setDT(TherapyRanges),  AgeInitiation,  AgeTermination)

  #finds ranges at which overlaps occur
  olaps.range<-foverlaps(TherapyRanges,VisitRanges,type="any",which=FALSE)
  olaps.min<-pmax(olaps.range$AgeatVisit.n1,olaps.range$AgeInitiation)
  olaps.max<-pmin(olaps.range$AgeatVisit.n2,olaps.range$AgeTermination)
  olaps.range<-data.frame(olaps.range)
```

```
  #finds indices at which overlaps occur
  olaps.id<-foverlaps(TherapyRanges,VisitRanges,type="any",which=TRUE)

  #locate indices of matched values and remove
  i.match<-olaps.min==olaps.max
  olaps.min<-olaps.min[!i.match]; olaps.max<-olaps.max[!i.match]

  #compute duration of overlaps and associated indices
  olaps.dur<-olaps.max-olaps.min
  olaps.id<-olaps.id[!i.match]

  #locate indices of low efficacy therapy
  i.low<-which(sheet3b.splitData[[i]]$Efficacy[olaps.id$xid] %in% 1)

  #locate indices of high efficacy therapy
  i.high<-which(sheet3b.splitData[[i]]$Efficacy[olaps.id$xid] %in% 2)

  #locate index of first therapeutic efficacy after first clinic visit
  i.future<-
c(which(sheet4.splitData[[i]]$AgeatVisit==intersect(sheet1.splitData[[i]]$Age,sheet4.splitData[[i]]$AgeatVi
sit)[1]))

  #now grab only efficacies from future therapies
  effcat.future<-
c(sheet3b.splitData[[i]]$Efficacy[olaps.id$xid][i.future:length(sheet3b.splitData[[i]]$Efficacy[olaps.id$xi
d])])

  #Factor as:
  #"None/Unknown" = no therapy (or unknown efficacy) after first visit
  #"Low Only" = only low efficacy therapy after first visit
  #"Both" = both low and high efficacy therapy after first visit
  #"High Only" = only high efficacy therapy after first visit
  logicals<-c(1,2) %in% effcat.future
  if(all(logicals==c("FALSE","FALSE"))=="TRUE"){eff.cat<-c(eff.cat,"None/Unknown")}
  if(all(logicals==c("TRUE","FALSE"))=="TRUE"){eff.cat<-c(eff.cat,"Low Only")}
  if(all(logicals==c("TRUE","TRUE"))=="TRUE"){eff.cat<-c(eff.cat,"Both")}
  if(all(logicals==c("FALSE","TRUE"))=="TRUE"){eff.cat<-c(eff.cat,"High Only")}

  #compute raw efficacy values for low and high efficacy therapy for each overlap period
  raw.lowEff<-lapply(i.low, fun.lowEff)
  raw.highEff<-lapply(i.high, fun.highEff)
```

```r
  #find positions of low and high efficacy and replace with raw values
  olaps.efficacy<-numeric(length(olaps.min))
  olaps.efficacy<-replace(olaps.efficacy,i.low,raw.lowEff)
  olaps.efficacy<-replace(olaps.efficacy,i.high,raw.highEff)

  #set all negative effiacies to zero
  olaps.efficacy[olaps.efficacy < 0] <- 0

  #Divide by 100 to convert raw to adjusted efficacy (percent to decimal)
  olaps.efficacy<- unlist(olaps.efficacy)/100

  #compute efficacy values (olaps.efficacy) for each treatment period (olaps.dur)
  olaps.efficacy<-split(olaps.efficacy,olaps.id$yid)
  olaps.dur<-split(olaps.dur,olaps.id$yid)

  #initialize lists
  col.count<-c()
  olaps.eff<-c()

  #divide durations into past (time from birth to first clinic visit) and
  #future (time from first clinic visit to last clinic visit)
  dur.past<-sheet1.splitData[[i]]$Age[1]
  dur.future<-sheet1.splitData[[i]]$Age[2:length(sheet1.splitData[[i]]$Age)]

  #compute efficacy of each therapy (product of efficacy and duration)
  eff<-Map(function(x,y) sum(x*y),unlist(olaps.efficacy),unlist(olaps.dur))

  #grab all efficacies up to first clinic visit
  eff.past<-eff[1:(nrow(sheet4.splitData[[i]])-nrow(sheet1.splitData[[i]]))]

  #grab all efficacies from first to last clinic visit
  eff.future<-eff[(length(eff.past)+1):length(eff)]

  #compute cumulative efficacies at first visit, last visit, and all future visits (between first and last
  #visit)
  first.cum.eff<-c(first.cum.eff,sum(unlist(eff.past))/first(sheet1.splitData[[i]]$Age))
  last.cum.eff<-c(last.cum.eff,sum(unlist(eff))/last(sheet1.splitData[[i]]$Age))
  future.cum.eff<-c(future.cum.eff,sum(unlist(eff.future))/(last(sheet1.splitData[[i]]$Age)-
first(sheet1.splitData[[i]]$Age)))
```

```
}

#add zero cumulative efficacy to untreated patients
first.cum.eff<-c(first.cum.eff,lapply(sheet1.omit.splitData, function(x) 0))
last.cum.eff<-c(last.cum.eff,lapply(sheet1.omit.splitData, function(x) 0))
future.cum.eff<-c(future.cum.eff,lapply(sheet1.omit.splitData, function(x) 0))

#add no/unknown to efficacy categorization for untreated patients
eff.cat<-c(eff.cat,lapply(sheet1.omit.splitData, function(x) "None/Unknown"))

#partition data by NIB number
sheet4.splitData<-split(sheet4,sheet4[1])

#----------------------------------------------------------------------------------------------------
#Compute CombiWISE unadjusted and adjusted slopes and EDSS slopes
#----------------------------------------------------------------------------------------------------

#conjoin lists of updated and omited patients
sheet1b.splitData<-c(sheet1.splitData,sheet1.omit.splitData)

#create empty list
rsquared<-c()

#loop through NIB subsets and compute slopes for CombiWISE and EDSS
for (i in 1:length(sheet1b.splitData)){

  #Unadjusted CombiWISE slope
  CW.lm.unadj<-lm(sheet1b.splitData[[i]]$CombiWISE ~ sheet1b.splitData[[i]]$Age)
  sheet1b.splitData[[i]]$CWslope.unadj<-coef(CW.lm.unadj)[2]
  sheet1b.splitData[[i]]$CWint.unadj<-coef(CW.lm.unadj)[1]
  rsquared<-c(rsquared,summary(lm(sheet1b.splitData[[i]]$CombiWISE ~
  sheet1b.splitData[[i]]$Age))$r.squared)

  #EDSS slope
  EDSS.lm<-lm(sheet1b.splitData[[i]]$EDSS ~ sheet1b.splitData[[i]]$Age, data=sheet1b.splitData[[i]])
  sheet1b.splitData[[i]]$EDSSslope<-coef(EDSS.lm)[2]

  #if measured CW slope is positive, then Adjusted CW slope = Measured CW Slope/(1-Eff(t0,tn)) where
  #t0 is the first clinic visit and tn is the last clinic visit
  if(sheet1b.splitData[[i]]$CWslope.unadj[1]>0){
    sheet1b.splitData[[i]]$CWslope.adj<-sheet1b.splitData[[i]]$CWslope.unadj/(1-future.cum.eff[[i]])
```

```
  #if measured CW slope is negative, then Adjusted CW Slope = Measured CW Slope*(1-Eff(t0,tn)) where
  #t0 is the first clinic visit and tn is the last clinic visit
  }else{sheet1b.splitData[[i]]$CWslope.adj<-(1-future.cum.eff[[i]])*sheet1b.splitData[[i]]$CWslope.unadj}

}

#-----------------------------------------------------------------------------------------------------
#Adjust first and last measured CombiWISE values for past therapy
#-----------------------------------------------------------------------------------------------------
for (i in 1:length(sheet1b.splitData)){
  sheet1b.splitData[[i]]$CombiWISE.adjusted.firstvisit<-first(sheet1b.splitData[[i]]$CombiWISE)/(1-
first.cum.eff[[i]])
  sheet1b.splitData[[i]]$CombiWISE.adjusted.lastvisit<-last(sheet1b.splitData[[i]]$CombiWISE)/(1-
last.cum.eff[[i]])
}

#-----------------------------------------------------------------------------------------------------
#1. Compute difference between adjusted CombiWISE and measured CombiWISE at first visit
#2. Append efficacy categorizations (aka "No/Unknown", "Low Only", "Both", "High Only") to dataframe
#3. Append cumulative efficacy computed from time period between first and last visit
#-----------------------------------------------------------------------------------------------------
for (i in 1:length(sheet1b.splitData)){
  sheet1b.splitData[[i]]$diff.adjCW.measCW<-sheet1b.splitData[[i]]$CombiWISE.adjusted.firstvisit-
first(sheet1b.splitData[[i]]$CombiWISE)
  sheet1b.splitData[[i]]$eff.cat<-eff.cat[i]
  sheet1b.splitData[[i]]$future.cum.eff<-future.cum.eff[i]
}

#-----------------------------------------------------------------------------------------------------
#Calculate time from disease onset to first DMT (prior to first clinic visit)
#-----------------------------------------------------------------------------------------------------

#for patients who received DMT
for (i in 1:length(sheet1.splitData)){

  #if age initiation occurs prior to first clinic visit, take difference between age at therapy initiation
and age at disease onset
  if (sheet2.splitData[[i]]$AgeInitiation[1]<=sheet1.splitData[[i]]$Age[1]){
    sheet1b.splitData[[i]]$Time2DMT.firstvisit<-sheet2.splitData[[i]]$AgeInitiation[1]-
as.numeric(sheet1.splitData[[i]]$AgeatDiseaseOnset[1])
```

```
  #if age initiation does not occur prior to first clinic visit, take difference between age at first visit
  #and age at disease onset
  }else{
    sheet1b.splitData[[i]]$Time2DMT.firstvisit<-sheet1.splitData[[i]]$Age[1]-
as.numeric(sheet1.splitData[[i]]$AgeatDiseaseOnset[1])
  }
}

#for patients who did not receive DMT
for (i in 1:length(sheet1.omit.splitData)){
  j=i+length(sheet1.splitData)

  #take difference between age at first visit and age at disease onset
  sheet1b.splitData[[j]]$Time2DMT.firstvisit<-sheet1.omit.splitData[[i]]$Age[1]-
as.numeric(sheet1.omit.splitData[[i]]$AgeatDiseaseOnset[1])
}

#-----------------------------------------------------------------------------------------------------
#Calculate time from disease onset to first DMT (prior to last clinic visit)
#-----------------------------------------------------------------------------------------------------

#for patients who received DMT
for (i in 1:length(sheet1.splitData)){

  #if age initiation occurs prior to last clinic visit, take difference between age of therapy initiation
  #and age at disease onset
  if (sheet2.splitData[[i]]$AgeInitiation[1]<=last(sheet1.splitData[[i]]$Age)){
    sheet1b.splitData[[i]]$Time2DMT.lastvisit<-sheet2.splitData[[i]]$AgeInitiation[1]-
as.numeric(sheet1.splitData[[i]]$AgeatDiseaseOnset[1])
  }

  #if age initiation does not prior to first clinic visit, take difference between age at last visit and
  #age at disease onset
  else{
    sheet1b.splitData[[i]]$Time2DMT.lastvisit<-last(sheet1.splitData[[i]]$Age)-
as.numeric(sheet1.splitData[[i]]$AgeatDiseaseOnset[1])
  }
}

#for patients who did not receive DMT
```

```
for (i in 1:length(sheet1.omit.splitData)){
  j=i+length(sheet1.splitData)

  #take difference between age at last visit and age at disease onset
  sheet1b.splitData[[j]]$Time2DMT.lastvisit<-last(sheet1.omit.splitData[[i]]$Age)-
as.numeric(sheet1.omit.splitData[[i]]$AgeatDiseaseOnset[1])
}

#--------------------------------------------------------------------------------------------------------
#Compute Measured CombiWISE/Age and Adjusted CombiWISE/Age at first and last clinic visit
#--------------------------------------------------------------------------------------------------------

Age<-unlist(sapply(sheet1b.splitData, function(x) x$Age)) #Age
CombiWISE<-unlist(sapply(sheet1b.splitData, function(x) x$CombiWISE)) #Measured CombiWISE
unadj.CombiWISE.first<-unlist(sapply(sheet1b.splitData, function(x) x$CombiWISE[1])) #Unadjusted CombiWISE
(first visit)
unadjCombiWISE.age.first<-unlist(sapply(sheet1b.splitData, function(x) x$CombiWISE[1]/x$Age[1]))
#Unadjusted CombiWISE/Age (first visit)
unadjCombiWISE.age.last<-unlist(sapply(sheet1b.splitData, function(x) last(x$CombiWISE)/last(x$Age)))
#Unadjusted CombiWISE/Age (last visit)
adjCombiWISE.age.first<-unlist(sapply(sheet1b.splitData, function(x)
x$CombiWISE.adjusted.firstvisit[1]/x$Age[1])) #Adjusted CombiWISE/Age (first visit)
adjCombiWISE.age.last<-unlist(sapply(sheet1b.splitData, function(x)
x$CombiWISE.adjusted.lastvisit[1]/last(x$Age))) #Adjusted CombiWISE/Age (last visit)

#--------------------------------------------------------------------------------------------------------
#Calculate global ARMSS
#--------------------------------------------------------------------------------------------------------
test_data<-data.frame("ageatedss"=unlist(sapply(sheet1b.splitData, function(x) x$Age)),
"edss"=unlist(sapply(sheet1b.splitData, function(x) x$EDSS)))
test_data_first<-data.frame("ageatedss"=unlist(sapply(sheet1b.splitData, function(x) x$Age[1])),
                            "edss"=unlist(sapply(sheet1b.splitData, function(x) x$EDSS[1])))
test_data_last<-data.frame("ageatedss"=unlist(sapply(sheet1b.splitData, function(x) last(x$Age))),
                            "edss"=unlist(sapply(sheet1b.splitData, function(x) last(x$EDSS))))
gARMSS<-global_armss(test_data)$data$gARMSS
gARMSS_first<-global_armss(test_data_first)$data$gARMSS
gARMSS_last<-global_armss(test_data_last)$data$gARMSS

#--------------------------------------------------------------------------------------------------------
#Export data as Excel spreadsheet
#--------------------------------------------------------------------------------------------------------
```

```
#Create blank columns for export purposes
for (i in 1:length(sheet1b.splitData)){
	sheet1b.splitData[[i]]$eff.cat<-
unlist(c(sheet1b.splitData[[i]]$eff.cat[1],rep("",nrow(sheet1b.splitData[[i]])-1)))
	sheet1b.splitData[[i]]$future.cum.eff<-
unlist(c(sheet1b.splitData[[i]]$future.cum.eff[1],rep("",nrow(sheet1b.splitData[[i]])-1)))
	sheet1b.splitData[[i]]$diff.adjCW.measCW<-
unlist(c(sheet1b.splitData[[i]]$diff.adjCW.measCW[1],rep("",nrow(sheet1b.splitData[[i]])-1)))
	sheet1b.splitData[[i]]$CombiWISE.adjusted.firstvisit<-
c(sheet1b.splitData[[i]]$CombiWISE.adjusted.firstvisit[1],rep("",nrow(sheet1b.splitData[[i]])-1))
	sheet1b.splitData[[i]]$CombiWISE.adjusted.lastvisit<-
c(sheet1b.splitData[[i]]$CombiWISE.adjusted.lastvisit[1],rep("",nrow(sheet1b.splitData[[i]])-1))
	sheet1b.splitData[[i]]$CWslope.unadj<-
c(sheet1b.splitData[[i]]$CWslope.unadj[1],rep("",nrow(sheet1b.splitData[[i]])-1))
	sheet1b.splitData[[i]]$CWslope.adj<-
c(sheet1b.splitData[[i]]$CWslope.adj[1],rep("",nrow(sheet1b.splitData[[i]])-1))
	sheet1b.splitData[[i]]$EDSSslope<-
c(sheet1b.splitData[[i]]$EDSSslope[1],rep("",nrow(sheet1b.splitData[[i]])-1))
	sheet1b.splitData[[i]]$MSSS.first<-
c(sheet1b.splitData[[i]]$MSSS[1],rep("",nrow(sheet1b.splitData[[i]])-1))
	sheet1b.splitData[[i]]$MSSS.last<-
c(last(sheet1b.splitData[[i]]$MSSS),rep("",nrow(sheet1b.splitData[[i]])-1))
	sheet1b.splitData[[i]]$Time2DMT.firstvisit<-
c(sheet1b.splitData[[i]]$Time2DMT.firstvisit[1],rep("",nrow(sheet1b.splitData[[i]])-1))
	sheet1b.splitData[[i]]$Time2DMT.lastvisit<-
c(sheet1b.splitData[[i]]$Time2DMT.lastvisit[1],rep("",nrow(sheet1b.splitData[[i]])-1))
	sheet1b.splitData[[i]]$TxUnder6.firstvisit<-
c(sheet1b.splitData[[i]]$TxUnder6.firstvisit[1],rep("",nrow(sheet1b.splitData[[i]])-1))
	sheet1b.splitData[[i]]$TxUnder6.lastvisit<-
c(sheet1b.splitData[[i]]$TxUnder6.lastvisit[1],rep("",nrow(sheet1b.splitData[[i]])-1))
	sheet1b.splitData[[i]]$CW.Age.unadj.firstvisit<-
c(unadjCombiWISE.age.first[i],rep("",nrow(sheet1b.splitData[[i]])-1))
	sheet1b.splitData[[i]]$CW.Age.unadj.lastvisit<-
c(unadjCombiWISE.age.last[i],rep("",nrow(sheet1b.splitData[[i]])-1))
	sheet1b.splitData[[i]]$CW.Age.adj.firstvisit<-
c(adjCombiWISE.age.first[i],rep("",nrow(sheet1b.splitData[[i]])-1))
	sheet1b.splitData[[i]]$CW.Age.adj.lastvisit<-
c(adjCombiWISE.age.last[i],rep("",nrow(sheet1b.splitData[[i]])-1))
	sheet1b.splitData[[i]]$gARMSS.first<-c(gARMSS_first[i],rep("",nrow(sheet1b.splitData[[i]])-1))
	sheet1b.splitData[[i]]$gARMSS.last<-c(gARMSS_last[i],rep("",nrow(sheet1b.splitData[[i]])-1))
```

```
       sheet1b.splitData[[i]]$AgeatDiseaseOnset<-
c(sheet1b.splitData[[i]]$AgeatDiseaseOnset[1],rep("",nrow(sheet1b.splitData[[i]])-1))
       sheet1b.splitData[[i]]$COMRIS.CTD.firstvisit<-
c(sheet1b.splitData[[i]]$COMRIS.CTD.firstvisit[1],rep("",nrow(sheet1b.splitData[[i]])-1))
       sheet1b.splitData[[i]]$COMRIS.CTD.lastvisit<-
c(sheet1b.splitData[[i]]$COMRIS.CTD.lastvisit[1],rep("",nrow(sheet1b.splitData[[i]])-1))
       sheet1b.splitData[[i]]$Gender<-
c(sheet1b.splitData[[i]]$Gender[1],rep("",nrow(sheet1b.splitData[[i]])-1))
       sheet1b.splitData[[i]]$fhxMS<-c(sheet1b.splitData[[i]]$fhxMS[1],rep("",nrow(sheet1b.splitData[[i]])-
1))
       sheet1b.splitData[[i]]$Smoke<-c(sheet1b.splitData[[i]]$Smoke[1],rep("",nrow(sheet1b.splitData[[i]])-
1))
       sheet1b.splitData[[i]]$Race<-c(sheet1b.splitData[[i]]$Race[1],rep("",nrow(sheet1b.splitData[[i]])-
1))
     }

 #unsplit list
 TreatedPts.splitData<-do.call("rbind", sheet1b.splitData[1:(length(sheet1b.splitData)-
length(sheet1.omit.splitData))])
 UntreatedPts.splitData<-do.call("rbind", sheet1b.splitData[(length(sheet1b.splitData)-
length(sheet1.omit.splitData)+1):length(sheet1b.splitData)])

 #data frame for treated patients
 TreatedPts.splitData<-data.frame(Patient.Code=toupper(TreatedPts.splitData$PatientCode),
                                  Cohort=TreatedPts.splitData$Cohort,
                                  Date.at.Visit=TreatedPts.splitData$Date,
                                  Age.at.Visit=TreatedPts.splitData$Age,
                                  Measured.CombiWISE=TreatedPts.splitData$CombiWISE,

Adjusted.CombiWISE.firstvisit=TreatedPts.splitData$CombiWISE.adjusted.firstvisit,

Adjusted.CombiWISE.lastvisit=TreatedPts.splitData$CombiWISE.adjusted.lastvisit,
                                  CW.Unadjusted.Slope=TreatedPts.splitData$CWslope.unadj,
                                  CW.Adjusted.Slope=TreatedPts.splitData$CWslope.adj,
                                  EDSS=TreatedPts.splitData$EDSS,
                                  EDSSslope=TreatedPts.splitData$EDSSslope,
                                  MSSS=TreatedPts.splitData$MSSS,
                                  MSSS.firstvisit=TreatedPts.splitData$MSSS.first,
                                  MSSS.lastvisit=TreatedPts.splitData$MSSS.last,
                                  Time2DMT.firstvisit=TreatedPts.splitData$Time2DMT.firstvisit,
                                  Time2DMT.lastvisit=TreatedPts.splitData$Time2DMT.lastvisit,
```

```
                                        TxUnder6.firstvisit=TreatedPts.splitData$TxUnder6.firstvisit,
                                        TxUnder6.lastvisit=TreatedPts.splitData$TxUnder6.lastvisit,
                                        CW.Age.unadj.firstvisit=TreatedPts.splitData$CW.Age.unadj.firstvisit,
                                        CW.Age.unadj.lastvisit=TreatedPts.splitData$CW.Age.unadj.lastvisit,
                                        CW.Age.adj.firstvisit=TreatedPts.splitData$CW.Age.adj.firstvisit,
                                        CW.Age.adj.lastvisit=TreatedPts.splitData$CW.Age.adj.lastvisit,
                                        global.ARMSS.first=TreatedPts.splitData$gARMSS.first,
                                        global.ARMSS.last=TreatedPts.splitData$gARMSS.last,
                                        Disease.Duration=TreatedPts.splitData$DiseaseDuration,
                                        Age.at.Disease.Onset=TreatedPts.splitData$AgeatDiseaseOnset,
                                        COMRIS.CTD.firstvisit=TreatedPts.splitData$COMRIS.CTD.firstvisit,
                                        COMRIS.CTD.lastvisit=TreatedPts.splitData$COMRIS.CTD.lastvisit,
                                        Gender=TreatedPts.splitData$Gender,
                                        fhxMS=TreatedPts.splitData$fhxMS,
                                        Smoke=TreatedPts.splitData$Smoke,
                                        Race=TreatedPts.splitData$Race,
                                        Diff.adjCombiWISE.measCombiWISE=TreatedPts.splitData$diff.adjCW.measCW,
                                        Cum.Eff.first2last=TreatedPts.splitData$future.cum.eff,
                                        Efficacy.categorization=TreatedPts.splitData$eff.cat,
                                        stringsAsFactors=FALSE)

#data frame for untreated patients
 UntreatedPts.splitData<-data.frame(Patient.Code=toupper(UntreatedPts.splitData$PatientCode),
                                        Cohort=UntreatedPts.splitData$Cohort,
                                        Date.at.Visit=as.character(UntreatedPts.splitData$Date),
                                        Age.at.Visit=UntreatedPts.splitData$Age,
                                        Measured.CombiWISE=UntreatedPts.splitData$CombiWISE,

Adjusted.CombiWISE.firstvisit=UntreatedPts.splitData$CombiWISE.adjusted.firstvisit,

Adjusted.CombiWISE.lastvisit=UntreatedPts.splitData$CombiWISE.adjusted.lastvisit,
                                        CW.Unadjusted.Slope=UntreatedPts.splitData$CWslope.unadj,
                                        CW.Adjusted.Slope=UntreatedPts.splitData$CWslope.adj,
                                        EDSS=UntreatedPts.splitData$EDSS,
                                        EDSSslope=UntreatedPts.splitData$EDSSslope,
                                        MSSS=UntreatedPts.splitData$MSSS,
                                        MSSS.firstvisit=UntreatedPts.splitData$MSSS.first,
                                        MSSS.lastvisit=UntreatedPts.splitData$MSSS.last,
                                        Time2DMT.firstvisit=UntreatedPts.splitData$Time2DMT.firstvisit,
                                        Time2DMT.lastvisit=UntreatedPts.splitData$Time2DMT.lastvisit,
                                        TxUnder6.firstvisit=UntreatedPts.splitData$TxUnder6.firstvisit,
```

```
                                      TxUnder6.lastvisit=UntreatedPts.splitData$TxUnder6.lastvisit,
                                      CW.Age.unadj.firstvisit=UntreatedPts.splitData$CW.Age.unadj.firstvisit,
                                      CW.Age.unadj.lastvisit=UntreatedPts.splitData$CW.Age.unadj.lastvisit,
                                      CW.Age.adj.firstvisit=UntreatedPts.splitData$CW.Age.adj.firstvisit,
                                      CW.Age.adj.lastvisit=UntreatedPts.splitData$CW.Age.adj.lastvisit,
                                      global.ARMSS.first=UntreatedPts.splitData$gARMSS.first,
                                      global.ARMSS.last=UntreatedPts.splitData$gARMSS.last,
                                      Disease.Duration=UntreatedPts.splitData$DiseaseDuration,
                                      Age.at.Disease.Onset=UntreatedPts.splitData$AgeatDiseaseOnset,
                                      COMRIS.CTD.firstvisit=UntreatedPts.splitData$COMRIS.CTD.firstvisit,
                                      COMRIS.CTD.lastvisit=UntreatedPts.splitData$COMRIS.CTD.lastvisit,
                                      Gender=UntreatedPts.splitData$Gender,
                                      fhxMS=UntreatedPts.splitData$fhxMS,
                                      Smoke=UntreatedPts.splitData$Smoke,
                                      Race=UntreatedPts.splitData$Race,

Diff.adjCombiWISE.measCombiWISE=UntreatedPts.splitData$diff.adjCW.measCW,
                                      Cum.Eff.first2last=UntreatedPts.splitData$future.cum.eff,
                                      Efficacy.categorization=UntreatedPts.splitData$eff.cat,
                                      stringsAsFactors=FALSE)

#bind data frames
sheet1b.splitData<-rbind(TreatedPts.splitData,UntreatedPts.splitData)

#reorder rows based on original input order
sheet1b.splitData[match(sheet1.df$PatientCode, sheet1b.splitData$Patient.Code),]

#reorder rows based on original input order
ids<-unlist(lapply(unique(sheet1.df$PatientCode), function(x) which(sheet1b.splitData$Patient.Code==x)))
sheet1b.splitData<-sheet1b.splitData[ids,]

#write to file
write.csv(sheet1b.splitData, file = "LongitudinalOuput_minuspeds_09222017.csv",row.names=F)
```

Script S2: R code for modeling MS-DSS using gradient boosting.

```
#-----------------------------------------------------------------
#Purpose: GBM model of MS-DSS
#-----------------------------------------------------------------
#Author: Chris Barbour, Montana State University & NIH
#E-mail: christopher.barbour@nih.gov, christopher.barbour@montana.edu
#Date: 5/26/2017
#-----------------------------------------------------------------

library(lubridate)
library(readr)
library(tidyr)
library(dplyr)
library(ggplot2)
library(purrr)
library(stringr)
library(gbm)
library(readxl)
library(bindrcpp)
library(snow)
library(rlecuyer)

# Puts Relative Influence from GBM into a nice tbl
gbm_imp <- function(gbm,ntree_method="cv",...){
  # gbm <- gbm_mod_both
  # ntree_method <- "cv"
  num_tree <- gbm.perf(gbm,plot.it = FALSE,method=ntree_method)
  toreturn <- summary(gbm,plotit=FALSE,n.trees=num_tree,...)
  toreturn <- toreturn %>%
    tbl_df() %>%
    mutate(var = as.character(var)) %>%
    mutate(var = str_replace_all(var,"`","")) %>%
    rename(relative_influence = rel.inf)
  return(toreturn)
}
```

```r
gbm_predstat <- function(gbm, x, y,plotit=TRUE,scientific=FALSE,digits=NULL,asp=NULL,pch=16,mult=1,
                         ci=TRUE,main="",...){
  pred <- predict.gbm(gbm,x,...)
  resid <- y - pred
  rmse <- sqrt(mean((y-pred)^2))
  full_y <- range(c(pred-mult*rmse,pred+mult*rmse))
  if(plotit==TRUE & ci==TRUE){
    # par(mfrow=c(1,2))
    plot(pred ~ y,xlab="Observed",ylab="Predicted",main=main,asp=asp,pch=pch,ylim=full_y);abline(0,1)
    segments(y,pred-mult*rmse,y,pred+mult*rmse)
    # plot(resid ~ pred,ylab="Residual",xlab="Predicted",pch=pch)
    # par(mfrow=c(1,1))
  }
  if(plotit==TRUE & ci==FALSE){
    plot(y~pred,ylab="Observed",xlab="Predicted",asp=asp,pch=pch,main=main);abline(0,1)
  }
  r_test <- cor.test(y,pred)
  r_pvalue <- r_test$p.value
  if(scientific == TRUE){r_pvalue <- format(r_pvalue,digits = digits,scientific=scientific)}
  return(tbl_df(data.frame(rmse = rmse,
                           r_pearson=as.numeric(r_test$estimate),
                           r_pvalue=r_pvalue)))
}

quant_bin<-function(x,quants=c(0,.5,1),...){
  quantiles<-quantile(x,quants)
  quantiles[1]<-quantiles[1]-0.001
  cut(x,breaks=quantiles,...)
}

flip_fac <- function(x){
  return(factor(x,levels=as.character(x)[length(x):1]))
}

fac_strip <- function(x){
  if(!(class(x) %in% c("character","factor"))){return(x)}
  if(class(x) %in% c("character","factor")){
    return(as.factor(as.character(x)))
  }
}
```

```r
char_2_fac <- function(x){
  if(class(x) == "character"){return(as.factor(x))}
  if(class(x) != "character"){return(x)}
}

fac_2_binary <- function(x){
  if(class(x) == "factor"){return(as.numeric(x) - 1)}
  else(return(x))
}

# Loading and cleaning the data provided by Ann
alldata_new <- read.csv("./data/raw/LongitudinalOuputforChris_minuspeds_09222017.csv",stringsAsFactors =
FALSE)
alldata_new <- alldata_new %>%
  rename(patient = Patient.Code,
         date = Date.at.Visit,
         age = Age.at.Visit,
         combi = Measured.CombiWISE,
         adj_combi_first = Adjusted.CombiWISE.firstvisit,
         adj_combi_last = Adjusted.CombiWISE.lastvisit,
         slope_adj = CW.Adjusted.Slope,
         onset_to_therapy_first = Time2DMT.firstvisit,
         onset_to_therapy_last = Time2DMT.lastvisit,
         num_6mo_treat_first = TxUnder6.firstvisit,
         num_6mo_treat_last = TxUnder6.lastvisit,
         comba_first = CW.Age.unadj.firstvisit,
         comba_last = CW.Age.unadj.lastvisit,
         adj_comba_first = CW.Age.adj.firstvisit,
         adj_comba_last = CW.Age.adj.lastvisit,
         disease_duration = Disease.Duration,
         age_at_onset = Age.at.Disease.Onset,
         comris_first = COMRIS.CTD.firstvisit,
         comris_last = COMRIS.CTD.lastvisit,
         gender = Gender,
         ms_fhx = fhxMS,
         smoke = Smoke,
         race = Race,
         adjustment_first = Diff.adjCombiWISE.measCombiWISE..at.first.visit.,
         cum_eff = Cum.Eff.first2last,
         ther_eff = Efficacy.categorization..therapy.received.between.first.and.last.visit.) %>%
```

```r
  mutate(comris_first = as.numeric(ifelse(comris_first %in% c("","na"),NA,comris_first)),
         comris_last = as.numeric(ifelse(comris_last %in% c("","na"),NA,comris_last))) %>%
  mutate(date = mdy(date)) %>%
  mutate(race = tolower(race),
         gender = tolower(gender),
         smoke = tolower(smoke),
         ms_fhx = tolower(ms_fhx)) %>%
  mutate(race = ifelse(race == "", "", ifelse(race == "white","white","non_white")),
         smoke = ifelse(smoke == "","", ifelse(smoke == "yes","yes","no_unknown")),
         ms_fhx = ifelse(ms_fhx == "","", ifelse(ms_fhx %in% c("no","unknown"),"no_unknown","yes"))) %>%
  group_by(patient) %>%
  mutate(first = ifelse(date == min(date),"yes","no"),
         last = ifelse(date == max(date),"yes","no")) %>%
  nest() %>%
  mutate(slope = sapply(data,function(dat){coef(lm(combi ~ age,data=dat))[2]})) %>%
  unnest() %>%
  ungroup

# Constructing dataset using observations from the first visit
baseline <- alldata_new %>%
  filter(first == "yes") %>%
  select(patient,slope_adj,age,combi, disease_duration, onset_to_therapy = onset_to_therapy_first,
         num_6mo_treat = num_6mo_treat_first, adj_comba = adj_comba_first, comris = comris_first,
         gender, ms_fhx, smoke, race,comba = comba_first, slope, adjustment_first, cum_eff, ther_eff)

add_comris <- read_excel("./data/raw/Additional_COMRIS_Data_06232017.xlsx")
add_comris <- add_comris %>%
  select(patient, comris) %>%
  arrange(patient)
baseline <- baseline %>%
  select(-comris) %>%
  merge(add_comris,by="patient") %>%
  select(patient,slope_adj,age,combi, disease_duration, onset_to_therapy,
         num_6mo_treat, adj_comba, comris,
         gender, ms_fhx, smoke, race,comba, slope, adjustment_first, cum_eff, ther_eff)

# Constructing dataset using observations from the last visit
last_cov <- alldata_new %>%
  filter(last == "yes") %>%
  select(patient,age,combi,disease_duration)
```

```
last_visit <- alldata_new %>%
  filter(first == "yes") %>%
  select(patient,slope_adj,onset_to_therapy = onset_to_therapy_last,
         num_6mo_treat = num_6mo_treat_last, adj_comba = adj_comba_last, comris = comris_last,
         gender, ms_fhx, smoke, race,comba = comba_last, slope,adjustment_first, cum_eff, ther_eff) %>%
  merge(last_cov,by="patient") %>%
  tbl_df() %>%
  select(patient,slope_adj,age,combi, disease_duration, everything())

# Training/Validation split - setting up the sampling groups
baseline <- baseline %>%
  mutate(cat_group = interaction(race,gender)) %>%
  group_by(cat_group) %>%
  mutate(comba_group = quant_bin(adj_comba),
         age_group = quant_bin(age)) %>%
  ungroup %>%
  mutate(comba_group = fac_strip(interaction(cat_group, comba_group)),
         age_group = fac_strip(interaction(cat_group, age_group)),
         sample_group = as.character(interaction(comba_group,age_group)))
baseline <- baseline %>%
  mutate(sample_group2 =
ifelse(str_detect(baseline$sample_group,"non_white")==TRUE,as.character(cat_group),sample_group)) %>%
  mutate(sample_group = as.factor(as.numeric(as.factor(sample_group2))))

# Training/Validation Split - Performing the split
set.seed(1085)
train_pats <- baseline %>%
  group_by(sample_group) %>%
  sample_frac(0.66) %>%
  ungroup %>%
  .$patient %>%
  as.character
baseline <- baseline %>%
  mutate(cohort = ifelse(patient %in% train_pats,"training","validation")) %>%
  select(-cat_group,-comba_group,-age_group,-sample_group,-sample_group2) %>%
  select(patient, slope_adj, cohort, everything())
last_visit <- last_visit %>%
  mutate(cohort = ifelse(patient %in% train_pats,"training","validation")) %>%
  select(patient, slope_adj, cohort, everything())

# Change characters to factors for running GBM models
```

```r
baseline <- baseline %>%
  mutate_all(funs(char_2_fac))
last_visit <- last_visit %>%
  mutate_all(funs(char_2_fac))

# Splits first visit data into training and validation datasets
training <- baseline %>%
  filter(cohort == "training") %>%
  select(-cohort)
testing <- baseline %>%
  filter(cohort == "validation") %>%
  select(-cohort)

# Splits last visit into training and validation datasets
last_train <- last_visit %>%
  filter(cohort == "training") %>%
  select(-cohort)
last_test <- last_visit %>%
  filter(cohort == "validation") %>%
  select(-cohort)

######################
# Using All Predictors
######################

# GBM Model Building

first_include <- names(training)[-c(1:2,5,14:15)]
set.seed(1085)
gbm_full <- gbm(training$slope_adj ~.,
                data=training[,first_include],
                distribution = "gaussian",
                n.trees = 5000, # 5000
                n.minobsinnode = 10, # 10
                interaction.depth = 2, # 2
                shrinkage = 0.001, # 0.001
                bag.fraction = 0.5, # 0.5
                cv.folds = 5, # 5
                keep.data = TRUE)
cviter <- gbm.perf(gbm_full,method="cv")
```

```r
# Predictions at first visit
gbm_predstat(gbm_full,training,training$slope_adj,n.trees=cviter,plotit=TRUE,
             scientific = TRUE,digits=4)
gbm_predstat(gbm_full,testing,testing$slope_adj,n.trees=cviter,plotit=TRUE,
             scientific = TRUE,digits=4)

# Predictions at last visit
gbm_predstat(gbm_full,last_train,last_train$slope_adj,n.trees=cviter,plotit=TRUE,
             scientific = TRUE,digits=4)
gbm_predstat(gbm_full,last_test,last_test$slope_adj,n.trees=cviter,plotit=TRUE,
             scientific = TRUE,digits=4)

# Relative Influence
gbm_imp(gbm_full)

# Important Variables
imp_var <- gbm_imp(gbm_full) %>%
  filter(relative_influence > 3) %>%
  .$var %>%
  as.character()


######################################
# Eliminate non-contributing variables
######################################

# Model Building
set.seed(1085)
gbm_red <- gbm(training$slope_adj ~.,
               data=training[,imp_var],
               distribution = "gaussian",
               n.trees = 5000,
               n.minobsinnode = 10,
               interaction.depth = 2,
               shrinkage = 0.001,
               bag.fraction = 0.5,
               cv.folds = 5,
               keep.data = FALSE)
cviter_red <- gbm.perf(gbm_red,method="cv")

# Predictions at first visit
```

```
gbm_predstat(gbm_red,training,training$slope_adj,n.trees=cviter_red,plotit=TRUE,
             scientific = TRUE,digits=4,asp=1,ci=FALSE)
gbm_predstat(gbm_red,testing,testing$slope_adj,n.trees=cviter_red,plotit=TRUE,
             scientific = TRUE,digits=4,asp=1,ci=FALSE)

# Predictions at last visit
gbm_predstat(gbm_red,last_train,last_train$slope_adj,n.trees=cviter_red,plotit=TRUE,
             scientific = TRUE,digits=4,asp=1,ci=FALSE)
gbm_predstat(gbm_red,last_test,last_test$slope_adj,n.trees=cviter_red,plotit=TRUE,
             scientific = TRUE,digits=4,asp=1,ci=FALSE)

# Relative Influence
gbm_imp(gbm_red)
```
